# Floral Scents and Fruit Aromas: Functions, Compositions, Biosynthesis, and Regulation

**DOI:** 10.3389/fpls.2022.860157

**Published:** 2022-03-10

**Authors:** Salma Mostafa, Yun Wang, Wen Zeng, Biao Jin

**Affiliations:** ^1^College of Horticulture and Plant Protection, Yangzhou University, Yangzhou, China; ^2^Department of Floriculture, Faculty of Agriculture, Alexandria University, Alexandria, Egypt

**Keywords:** flower scent, fruit aroma, volatile function, VOC, biosynthetic pathway, transcription factor

## Abstract

Floral scents and fruit aromas are crucial volatile organic compounds (VOCs) in plants. They are used in defense mechanisms, along with mechanisms to attract pollinators and seed dispersers. In addition, they are economically important for the quality of crops, as well as quality in the perfume, cosmetics, food, drink, and pharmaceutical industries. Floral scents and fruit aromas share many volatile organic compounds in flowers and fruits. Volatile compounds are classified as terpenoids, phenylpropanoids/benzenoids, fatty acid derivatives, and amino acid derivatives. Many genes and transcription factors regulating the synthesis of volatiles have been discovered. In this review, we summarize recent progress in volatile function, composition, biosynthetic pathway, and metabolism regulation. We also discuss unresolved issues and research perspectives, providing insight into improvements and applications of plant VOCs.

## Introduction

Floral scents and fruit aromas are volatile organic compounds (VOCs) released by plants. These VOCs are lipophilic and characterized by low molecular weights and high melting points. Biosynthesis of VOCs occurs in all plant organs, including seeds, roots, stems, leaves, fruits, and flowers; floral scents and fruit aromas are relevant to our daily lives and have substantial economic value.

Floral VOCs are important traits of floral scents. The differences and abundances of these VOCs in scents vary widely among flowering plants (Farré-Armengol et al., 2017). According to their origin, biosynthesis, and function, floral scents are classified as terpenoids, phenylpropanoids, fatty acid derivatives and amino acids.

Fruit aroma is a key contributor to fruit quality and acceptance by animal and human. Fruit aroma consists of various chemical compounds (e.g., aldehydes, alcohols, ketones, esters, lactones, and terpenes) (Riu-Aumatell et al., 2004); the presence or absence of certain compounds determines differences among fruit aromas. In a wide range of fruits, the production of volatile compounds beginning from fruit-set to late-ripening is modulated by the accumulation of fruity esters, terpenes, and other compounds.

In the past decade, numerous studies of floral scents and fruit aromas have improved our understanding of their functions, components, biosynthesis, and regulation. In addition, previous reviews focused on floral scents or fruit aromas separately, whereas both have important volatile components contributing to the economic value of horticultural plants. Therefore, we review recent progress regarding floral scents and fruit aromas, focusing on the functions and compositions of volatiles, the factors and biosynthetic pathways that affect volatiles, and the regulation of their biosynthesis and metabolism.

## Functions

### Floral Scent Volatiles

Floral volatiles attract effective pollinators for sexual reproduction (Schiestl, 2010; Gross et al., 2016). Although some floral VOCs attract a variety of pollinators (broad pollinators) (Schiestl et al., 2003), flowers release volatiles to signal to specific pollinators (Schiestl, 2015). Their selection behavior depends on differences in composition, amount, and emission of floral volatile compounds (Muhlemann et al., 2014). For instance, emitted terpenoids and benzenoids can attract pollinators and repel elective visitors (Farré-Armengol et al., 2013), while the same terpenoid compound may attract one animal but repel another (Eilers et al., 2021). Many flowers offer floral rewards as nectar, pollen or oil products, on which visitors depend (Steiner et al., 2011) but some plants attract pollinators without offering nectar by mimicking the scents and colors of neighboring plants (Kunze, 2001; Schiestl, 2005). High production of VOCs is necessary to guide pollinators to flowers, particularly to overcome the decreased conspicuousness of flowers at night (Muhlemann et al., 2014). β-ocimene is implicated in the attraction of pollinators. For example, in *Mirabilis jalapa* flowers, the emitted floral scent is dominated by *trans-*β-ocimene and its emission peaks in the evening, which match flower opening and hawkmoth pollinator activity (Effmert et al., 2005).

Other adaptive roles for floral scents include repellents (Piechulla and Pott, 2003; Kessler et al., 2008; Raguso, 2008) and the provision of physiological protection against biotic stresses in plants (Dudareva et al., 2006; Knudsen and Gershenzon, 2006). In plant allelopathy, different VOCs can function as cues to other plants (Caruso and Parachnowitsch, 2016). For instance, in plant-plant communication, plants use volatiles emitted by their neighbors to evaluate their environment, including the presence of herbivores (Heil and Karban, 2010) and competitors (Kegge and Pierik, 2010). Generally, exposure of higher plants to biotic stresses (e.g., herbivore damage) can result in the emission of VOCs to repulse the attack (Arimura and Pearse, 2017) and enhance the response to future attacks (Morrell and Kessler, 2014). Similarly, some flower VOCs can be used in plant allelopathy. For example, the olfactory cues of some non-host plants attract the pollinators of other plants (e.g., *Malva moschata* and *Geranium sanguineum* attract *Chelostoma rapuncul* bees, the pollinator of *Campanula* spp.) (Burger et al., 2021). However, the compositions of floral scents that attract pollinators or repel herbivores are unclear.

Some volatiles have antibacterial properties and function in defense against microbial pathogens (Junker et al., 2011; Huang et al., 2012). Certain defensive functions rely on a single compound, which is more effective than a mixture of compounds (Gershenzon and Dudareva, 2007). A single floral volatile can have multiple roles in flowers. For example, (E)-b-caryophyllene from *Arabidopsis thaliana* promotes defense against pathogens and enhances pollinator attraction (Huang et al., 2012). In rose geranium (*Pelargonium graveolens*), β-citronellol is an abundant compound with extensive antibacterial activity (Boukhris et al., 2015). The reported functions of floral scents are listed in Table 1.

**TABLE 1:** Functions of floral scents.

Function	Species	References
Pollinator attraction	In most species, for example Orchid (*Catasetum arietinum*)	Brandt et al., 2020
	Peony (*Paeonia brownie*)	Bernhardt et al., 2013
	*Mirabilis jalapa*	Effmert et al., 2005
Protection against abiotic stresses (Low or high temperature, drought, salinity)	*Osmanthus fragrans*	Fu et al., 2017
	*Petunia axillaris*	Sagae et al., 2008
Protection against biotic stresses (Defense against bacteria, fungus and other micro-organisms)	Rose geranium (*Pelargonium graveolens)*	Boukhris et al., 2015
	*Salvia hydrangea*	Ghavam et al., 2020
	*Jasminum sambac*	Santhanam et al., 2014
	*Rosa damascene*	Shohayeb et al., 2014
	*Pelargonium graveolens*	Androutsopoulou et al., 2021
Floral cues to other plants	*Solidago altissima*	Kalske et al., 2019
Floral cues encourage visits to non-host plants	*Malva moschata* and *Geranium sanguineum*	Burger et al., 2021

### Fruit Aroma Volatiles

Fruit aroma is a major contributor to fruit quality (color, texture, flavor, and aroma). In the wild, the aroma VOCs released from fruits influence herbivore behavior and attract animal dispersers (Dudareva et al., 2013). For example, fruit bats recognize ripe and non-ripe fruits based on the emitted volatiles (Hodgkison et al., 2007). VOCs have biological activities against bacteria, fungi, and insects. Volatiles extracted from citrus peels (*Citrus reticulata* Blanco) exhibit significant antifungal and antibacterial activities against pathogenic strains (Sultana et al., 2012). In another citrus species (*Citrus hystrix*), the essential oils extracted from fruit peels possess antibacterial activity against respiratory bacteria; the most effective components are α-terpineol, terpinene-4-ol, and limonene (Srisukh et al., 2012). The antibacterial activities of fruit VOCs in extracts of lemon (*Citrus limonium*), sweet lime (*Citrus limetta*), pomegranate (*Punica granatum*), apple (*Malus domestica*), and tomato (*Solanum lycopersicum*) against pathogenic bacteria isolated from a wound have been reported (Unnisa et al., 2012). Essential oils extracted from *Alchornea cordifolia* fruit have shown antibacterial activity against *Staphylococcus aureus* and antifungal activity against *Aspergillus niger*, while essential oils extracted from *Canthium subcordatum* fruit have shown antibacterial activity against *Bacillus cereus*, *S. aureus*, and *A. niger* (Essien et al., 2015). In strawberry fruit, the antifungal activity of fruit VOCs against *Colletotrichum acutatum* is contributed by (E)-hex-2-enal (Arroyo et al., 2007). Essential oil components from pepper fruit significantly inhibited the germination of *Colletotrichum gloeosporioides*; the active compounds were carvacrol, cinnamon oil, citral, *trans*-cinnamaldehyde, *p*-cymene, and linalool (Hong et al., 2015). Volatiles isolated from leaves, flowers, and fruits of three opuntia species had antifungal activity against fungal species such as *Alternaria solani* (Bergaoui et al., 2007). Although fruit VOCs have antibacterial and antifungal activities, the most important function of fruit aroma in horticulture is to attract humans. Indeed, fruit aromas have been selected by horticulture breeding to improve their edible quality and economic value.

## Volatile Composition

### Floral Scents

Floral scents consist of a mixture of compounds; they are categorized as terpenoids, phenylpropanoids/benzenoids, fatty acids, and amino acids. Terpenoids are the largest class of volatiles and comprise more than 40,000 structures derived from five-carbon isoprene units as monoterpenoids, sesquiterpenoids, apocarotenoids, and others. Phenylpropanoids comprise more than 8,000 metabolites (Dong and Lin, 2021); benzenoids are the second class of VOCs derived from the amino acid phenylalanine. Fatty acids and amino acids are important VOCs present in floral scents and fruit aromas (Negre-Zakharov et al., 2009).

#### Rose (*Rosa* spp.)

Rose is one of the most important ornamental plants for the production of cut flowers and perfumes. Therefore, floral scent is the main economic trait of rose, and some cultivars are classified according to their fragrant components (Du et al., 2019). Rose scents are composed of mixtures of organic compounds. In Bulgarian rose (*Rosa damascene*) essential oil, the most abundant components are phenethyl alcohol, citronellol, heneicosane, pentadecane, eugenol, methyleugenol, and geraniol (Won et al., 2009; Kiralan, 2015; Koksal et al., 2015). The first rose water (*R. damascene*) comprised benzoic acid 2-hydroxy-3-methyl butyl ester, geranyl acetate, carbamic acid methyl ester, linalool, eucalyptol, citronellol, geraniol, and methyleugenol (Koksal et al., 2015). In other rose (*Rosa* L.) species (*R. dumalis*, *R. canina*, *R. dumalis subsp. boissieri*, *R. gallica*, and *R. hirtissima*), the major compounds are aldehydes, alcohols, monoterpenes, and sesquiterpenes (Demir et al., 2014). Moreover, the fragrant rose cultivar “Fragrant Cloud” contains monoterpene alcohols, terpene hydrocarbons, and acetates (Shalit et al., 2004). The predominant scent compounds in the petals of six Hybrid Rugosa roses are phenyl ethyl alcohol, β-citronellol, nerol, and geraniol (Sparinska and Rostoks, 2015). Rose-breeding programs are underway to produce new varieties of floral scents.

#### Orchid

Orchid is the largest family (Orchidaceae) of flowering plants (Julsrigival et al., 2013), comprising 20,000–30,000 species (Khuraijam et al., 2017). Approximately 75% of orchids are fragrant (De et al., 2019). In an economic context, they can be used to produce cut flowers and as potted plants (Khuraijam et al., 2017). The family Orchidaceae is divided into five subfamilies. Epidendroideae is the largest subfamily, comprising approximately 76% of the family (Freudenstein and Chase, 2015). In Epidendroideae, the major aromatic compounds of Thai fragrant orchid species (*Rhynchostylis gigantea* Ridl., *R. gigantea* var. *harrisonianum* Holtt., *Vanda coerulea*, and *Dendrobium parishii* Rchb. f.) are nerol, 2,3-dihydrofarnesol, nonanal, and 2-pentadecanone (Julsrigival et al., 2013). The emitted volatiles of *Zygopetalum maculatum* (an orchid species) are enriched in benzenoids, including *O*-diethylbenzene, p-diethylbenzene, benzyl acetate, and methyl salicylate; 2-phenylethylacetate is the major phenylpropanoid component (Bera et al., 2018). *Cymbidium* spp. flowers are rich in cineole, isoeugenol, and (-) selinene (Ramya et al., 2020). *Phalaenopsis* orchids contain monoterpenes, including linalool and geraniol (Chuang et al., 2017). The scent compounds in Vanda Mimi Palmer (an orchid hybrid) are ocimene, linalool, linalool oxide, and nerolidol as the major terpenoids; the major benzenoids and phenylpropanoids are benzyl acetate, methylbenzoate, phenylethyl acetate, and phenylethanol (Mohd-Hairul et al., 2010). The major components of the floral scents of *Ophrys sphegodes*, *Ophrys bertolonii*, and *Neotinea tridentate* are hydrocarbons, alcohols, aldehydes, and terpenes (Manzo et al., 2014).

#### Tulip (*Tulipa* spp.)

The few fragrant tulip cultivars produce a range of floral scents (Oyama-Okubo and Tsuji, 2019). The major scent compounds of tulips (*Tulipa L*.) cultivars are monoterpenoids (eucalyptol, d-limonene, linalool, *trans-*β-ocimene, and α-pinene), sesquiterpenoids (α-farnesene, caryophyllene, geranyl acetone, and β-ionone), benzenoids (benzaldehyde, acetophenone, 3,5-dimethoxytoluene, benzyl alcohol, methyl salicylate, and 2-phenyl ethanol), and fatty acid derivatives (decanal, *cis-*3-hexenol, *cis-*3-hexenyl acetate, 2-hexenal, and octanal) (Oyama-Okubo and Tsuji, 2013).

#### Peony (*Paeonia* spp.)

Peony (tree and herbaceous peony) includes many Paeoniaceae species and cultivars, which are important ornamental plants with rich fragrances. The major flavoring substances in peony are citronellol, geraniol, and linalool (Song et al., 2018; Wang et al., 2021). In 30 herbaceous peony cultivars, the main compounds are phenyl ethyl alcohol, β-caryophyllene, linalool, nerol, and (R)-citronellol (Song et al., 2018).

#### Lily (*Lilium* spp.)

Lilies are commercially important ornamental plants because of their attractive colors and scents (Du et al., 2019). Volatile-emitting lily cultivars (*Lilium* spp.) release various scent compounds, predominantly three monoterpenoids (1.8-cineole, (E)-b-ocimene and linalool) and one benzenoid (methyl benzoate) (Kong et al., 2017). β-*cis-*ocimene represents the major component in *Lilium* spp. “Sweetness” (Aros et al., 2020).

#### Water Lily (Family: Nymphaeaceae)

Water lily flowers (*Nymphaea colorata*) release 11 volatiles, comprising terpenoids (sesquiterpenes), fatty acid derivatives (methyl decanoate), and benzenoids (Zhang et al., 2020).

#### Carnation (*Dianthus* spp.)

Benzenoids are the principal components of carnation flowers in addition to terpenoids and fatty acid derivatives (Kishimoto, 2020). Some scents are described as spicy because they contain eugenols (Kishimoto, 2020). In *Dianthus rupicola* Biv. (cliffs carnation), phenolic monoterpenes are the predominant components of the essential oil, followed by monoterpene hydrocarbons and oxygen-containing sesquiterpenes (Casiglia et al., 2014). In a carnation species (*D. elymaiticus*), the essential oil of flowers contains high levels of fatty acid derivatives and terpenoids; the major compounds are (Z)-3-hexenyl acetate, methyl benzoate, β-caryophyllene, and decanal (Azadi and Entezari, 2016).

#### Lavender (*Lavandula* spp.)

Lavender plants have excellent medicinal and aromatic properties (Guo and Wang, 2020). The major volatiles extracted from lavender essential oil (*Lavandula angustifolia* Mill) are linalool, linalyl acetate (Venskutonis et al., 1997; Won et al., 2009; Guo and Wang, 2020), 1,8-cineole, and α-terpineol (Tschiggerl and Bucar, 2010; Śmigielski et al., 2013), along with oxygenated derivatives of monoterpenes and monoterpene alcohols (Śmigielski et al., 2013). In *L. officinalis*, linalyl acetate was the predominant volatile in the essential oils (Xiao et al., 2017). Lavender flower (*L. angustifolia*) vapor consists of terpene hydrocarbons, oxygenated terpenes, and sesquiterpenes; linalool, terpin-4-ol, and linalyl acetate are predominant (An et al., 2001).

#### Jasmine (*Jasminum* spp.)

Jasmine flowers are well-known for their pleasant fragrances. In *Jasminum* species (*J. sambac*, *J. auriculatum*, *J. grandiflorum*, and *J. multiflorum*), linalool and (3E, 6E)-a-farnesene are the major monoterpene and sesquiterpene, respectively (Bera et al., 2015). *J. sambac* is a fragrant flower species distributed worldwide. The major compounds of the floral scent are methyl anthranilate, linalool, 4-nonanolide, 4-hexanolide, (E)-2-hexenyl hexanoate, 4-hydroxy-2,5-dimethyl-3(2 H)-furanone (Ito et al., 2002), eugenol, benzyl alcohol, benzyl acetate, benzyl benzoate, methyl salicylate, methyl anthranilate, (Z) 3-hexenyl benzoate, indole, and α-farnesene (Lin et al., 2013; Chen et al., 2017, 2020).

#### Daffodil (*Narcissus* spp.)

Narcissus floral volatiles and their essential oils are used in the perfume industry (Terry et al., 2021). Chinese daffodil flowers (*Narcissus tazetta*) contain acetic acid phenethyl ester, E-ocimene, acetic acid benzyl ester, neo-allo-ocimene, allo-ocimene, α-linalool, 1,8 cineole, benzenepropyl acetate, and 3-methyl-2-buten-1-ol acetate as the major VOCs (Melliou et al., 2007; Song et al., 2007; Chen et al., 2013). Monoterpenes are the major VOCs in daffodil (*N. pseudonarcissus*); β-ocimene and β-myrcene are predominant (Li et al., 2018). In simple and double flower cultivars of *Narcissus*, the major scent compounds are monoterpenes and benzenoids, particularly *cis-*β-ocimene and benzyl acetate (Ruíz-Ramón et al., 2014). In the essential oil of *N. serotinus*, the major component is benzyl acetate; linalool oxides are the main characteristics of this species (Melliou et al., 2007). In two varieties of European Daffodil (*N. pseudonarcissus* L.), “Isha” and “Acropolis,” the major categories of VOCs are terpenes and ethers; the characteristic aromatic component is β-ocimene (Wang et al., 2013).

#### Hyacinth (*Hyacinths* spp.)

In Hyacinth (*Hyacinths orientals*) varieties, the major components of floral scents are terpenes, esters, and alcohols; the characteristic aromatic components are acetoxytoluene, β-ocimene, β-myrcene, and β-phenethyl alcohol (Wang et al., 2013).

Most studies of floral scents have focused on VOC profiles. However, the components of specific species (cultivars) should be evaluated by mass spectrometry and a sensory evaluation of floral fragrances. The chemical compositions of floral scents of major flower species are listed in Supplementary Table 1.

### Fruit Aroma

According to the modes of fruit development, there are three main groups (botanical classification): simple fruits, aggregate fruits, and multiple (or composite) fruits (Singh, 2004). However, based on the yield and commercial values, the fruits are normally classified into berries, melons, citrus fruits, drupes (stone fruits), pomes (apples and pears), and tropical fruits. Most fruits release a wide range of VOCs, which determine their aroma profiles. The VOCs released from fruits are esters, ketones, aldehydes, lactones, alcohols, and terpenoids (Ahmed et al., 2013). C_10_ monoterpenes and C_15_ sesquiterpenes are abundant and key determinants of the characteristic aroma of fruits (Ahmed et al., 2013). Each fruit species has a distinctive aroma based on the mixture of fruit VOCs (Tucker, 1993; Baietto and Wilson, 2015).

#### Banana (*Musa* spp.)

Banana is the main fruit traded globally (Alam, 2014). Almost 200 volatile components are found in banana fruit (Alam, 2014). Hexanal is the major volatile compound in most banana cultivars. The typical volatile compounds are (*E*)-2-hexenal and acetoin (Cavendish), (*E*)-2-hexenal and hexanal (Plantain), and 2,3-butanediol (Frayssinette) (Aurore et al., 2011). In addition, VOC composition may change during fruit maturation. During the ripening of two banana cultivars (“Brazilian” and “Fenjiao”), the predominant volatile components are isoamyl acetate, butanoic acid, 3-methyl-3-methylbutyl ester, hexanal, *trans-*2-hexenal, and 1-hexanol. However, octanoic acid and propanoic acid 2-methylbutyl ester are only detected in Fenjiao (Zhu et al., 2018a).

#### Apple (*Malus domestica*)

More than 300 aromatic compounds have been identified in apples, including esters, alcohols, aldehydes, acids, ketones, and terpenoids (Yang S. et al., 2021). Esters are the most important determinant of the aroma of ripe apples, followed by alcohols (Espino-Díaz et al., 2016). Although the aroma is cultivar-specific, eight common VOCs are detected in 40 apple cultivars: esters (hexyl butyrate, hexyl 2-methylbutyrate and hexyl hexanoate), hexanal, (E)-2-hexenal, 1-hexanol, estragole, and α-farnesene (Yang S. et al., 2021). In addition, esters such as butyl acetate, hexyl acetate, and 2-methyl butyl acetate influence the aroma profiles of the apple cultivars “Discovery” and “Prima” (Dunemann et al., 2009). There are more than 7,500 cultivars of culinary and eating apples (Elzebroek and Wind, 2008); thus, more aroma components must be identified.

#### Grape (*Vitis vinifera*)

Grape is of great commercial significance worldwide; it is divided into table (fresh consumption), juice, wine, and dried (raisins) types. The components of three varieties (Merlot, Cabernet Sauvignon, and Feteasca Neagra) of wine grape are: butanoic acid, tropilidene, methyl ester, 2-ethyl heptanoic acid, 2,4-dimethyl heptane, 2,4-dimethyl-1-heptene, *n-*nonane, 4-methyl octane, 2-propyl-1-pentanol, 6-methyl tridecane, 3,5-dimethyl octane, *n-*decane, *O*-cymene, terpinen-4-ol, undecane, linalool, and estragole (Palade and Popa, 2016). During development of muscadine grape (*Vitis rotundifolia*), myrcenol, β-ocimene, and L-limonene are common components at the first stage (green). Nonanal, decanal, and β-citronellol are detected at the second stage (soft and translucent and skin pink/red); at the third stage (purple to black), butyl-2-butenoate, propyl acetate, hexyl acetate, hexyl-2-butenoate, ethyl *trans-*2-butenoate, ethyl acetate, 1-octanol, butyl acetate, ethyl hexanoate, and β-citral are detected (Lee et al., 2016). The most common VOCs in white “Albariño” grapes are (E)-2-hexenal, (Z)-2-hexanol, 1-hexanol, benzaldehyde, phenylethanal, 2-phenylethanol, and *cis* pyran linalool oxide (Ripoll et al., 2017). In table grape cultivars (Centennial Seedless, Italia, Italia Rubi, Chasselas, Alphonse Lavallée, and Muscat de Hambourg), (E)-2-hexenal and hexanal are the two major volatiles, whereas monoterpenols are specific to Muscat varieties (Aubert and Chalot, 2017).

#### Strawberry (*Fragaria* spp.)

Several compounds are found at high concentrations (such as ketones and long-chain acids) in VOCs of strawberry, whereas several characteristic VOCs such as furanones (particularly 4-methoxy-2,5-dimethyl-3(2H)-furanone), esters (ethyl butanoate, ethyl hexanoate, methyl butanoate, and methyl hexanoate), terpenes (linalool and nerolidol) and sulfur compounds (methanethiol) are present at low levels (Yan et al., 2018). In white strawberry (*Fragaria chiloensis*), the major aroma compounds are ethyl butanoate, 2-hexenal, ethyl hexanoate, hexyl acetate, 2-hexen-1-ol acetate, furfuryl acetate, linalool, mesifuran, ethyl decanoate, benzyl alcohol, 2-phenylethyl acetate, 2,5-dimethyl-4-hydroxy-3(2H)-furanone, hydrocinnamyl alcohol, γ-decalactone, cinnamyl acetate, (E)-2,6-dimethylocta-2,7-dien-1,6-diol, (Z)-2,6-dimethylocta-2,7-dien-1,6-diol, and hexadecanoic acid (Prat et al., 2013).

#### Citrus (*Citrus* spp.)

Citrus fruits (e.g., orange and lemon) have high nutritional and economical value because of their contents of vitamin C, flavonoids, pectin, carotenoids, and calcium (Abobatta, 2019). Global production reached 98 million tons in 2021, according to the United States Department of Agriculture. The aromatic compounds in citrus fruits are present in peels and juices; their essential oils are used in the food, cosmetics, and pharmaceutical industries. Terpenes and terpenols are the major volatiles in orange juice. In orange beverage, limonene is the main volatile compound, followed by myrcene, ethyl butyrate, γ-terpinene, linalool, 3-carene, decanal, ethyl acetate and low levels of 1-octanol, geranial, β-pinene, octanal, α-pinene, and neral (Mirhosseini et al., 2007). Similarly, the main aromatic compounds in orange essence oil (from juice) are limonene followed by linalool, octanal, decanal, myrcene, and ethyl butyrate (Högnadóttir and Rouseff, 2003). In sweet orange (*Citrus sinensis*) juice, ethyl butanoate, nootkatone, linalool, and limonene are predominant (Kelebek and Selli, 2011; Herrera et al., 2016); the aroma profile of Jinchen sweet orange juice and peel oil comprises the same VOCs (Qiao et al., 2008).

The volatile oil of *Citrus limon* peels consists mainly of monoterpenes; limonene is the most abundant component (Ayedoun et al., 1996; Mahalwal and Ali, 2003), followed by camphenes, α-terpineol, α-phellandrene, and 4-terpineol, along with α-selinene (a predominant sesquiterpene), caryophyllene oxide, t-nerolidol, and valencene (Mahalwal and Ali, 2003). In eureka lemon, terpenoids are the main aromatic components; d-limonene is the major component in lemon juice and peel, followed by aldehydes and esters (Zhong et al., 2014).

#### Mango (*Mangifera indica*)

Mango is a tropical fruit and a good source of vitamins, minerals, and fiber. According to the latest report of global fruit production in 2019^1^, mango ranked sixth among the major fruits. The predominant aroma compounds in mango are (E)-β-damascenone, (E,Z)-nonadienal, (E)-2-nonenal, (E)-β-ionone, terpinolene, ethyl 2-methylpropanoate, ethyl butanoate, ethyl 2-methylbutanoate, limonene, myrcene, linalool, δ-3-carene, β-caryophyllene, γ-octalactone, nonanal, methyl benzoate, 2,5-dimethyl-4-methoxy-3(2H)-furanone, and hexanal (Pino, 2012). In addition to ethyl-2-methylpropanoate, ethyl butanoate, and methyl benzoate, the most abundant aromatic compounds in 20 mango cultivars are (E)-2-nonenal, (E,Z)-2,6-nonadienal, decanal, (E)-β-ionone, and 2,5-dimethyl-4-methoxy-3(2H)-furanone (Pino and Mesa, 2006). Some cultivars have typical aromas; examples include Colombian mangoes (α-pinene, α-phellandrene and terpinolene) (Quijano et al., 2007) and Harumanis mango (β-ocimene, *trans*β-ocimene, and allo-ocimene) (Zakaria et al., 2018). Mango skin produces glycosidically bound aromatic volatile compounds, the levels of which are strongly influenced by fruit part and maturity (Lalel et al., 2003a).

#### Peach (*Prunus persica*)

Peach is an important stone fruit; it contains vitamins, minerals, and sugars. The volatile levels in white-fleshed peach skin are significantly higher than in other parts of the fruit. Unsaturated lactones and C6-compounds are detected mainly in the top and bottom mesocarp; benzaldehyde content is highest close to the stone (Aubert and Milhet, 2007). The essential oils of six peaches comprise aldehydes, lactones, alcohols, terpenes, esters, acids, norisoprenoids, phenylalanine derivates, and ketones (Eduardo et al., 2010). The characteristic volatiles and their contents in peach cultivars depend on the genotypic background and germplasm origin. For example, the highest contents of terpenoids and esters are present in Chinese wild peaches and “Wutao,” lactones are present in “Ruipan 14” and “Babygold 7,” and linalool is present in seven cultivars of American or European origin (Wang et al., 2009).

#### Apricot (*Prunus armeniuca*)

The major volatiles in apricot are aldehydes, alcohols, esters, acetates, terpenes, and acids. The most abundant compounds are ethanol, hexanal, hexyl acetate, (Z)-3-hexenyl acetate, (E)-2-hexenyl acetate, (Z)-3-hexenol, 1-hexanol, and (E)-2-hexen-1-ol (Gokbulut and Karabulut, 2012). The major compounds in other apricot species are heptyl isobutyrate, citronellyl propionate, geranyl acetate, γ-hexalactone, δ-undecalactone, 5-hydroxy-7-decenoic acid lactone, and 5-hydroxy-2,4-decadienoic acid lactone (Zhang et al., 2008). In some apricot cultivars, ethyl acetate, hexyl acetate, limonene, β-cyclocitral, γ-decalactone, 6-methyl-5-hepten-2-one, linalool, β-ionone, menthone, and (E)-hexen-2-al are the most important aromatics (Guillot et al., 2006). In the apricot cultivar “Xinshiji,” the predominant compounds are hexyl acetate, β-ionone, butyl acetate, linalool, limonene, γ-decalactone, (E)-2-hexenal, and hexanal (Meixia et al., 2004; Chen et al., 2006). Aldehydes and terpenes decrease significantly, whereas lactones and apocarotenoids increase significantly, with apricot ripening. β-ionone, γ-decalactone, sucrose, and citrate are key flavor characteristics influencing consumer acceptance (Xi et al., 2016). In ripe “Xinshiji” and “Hongfeng” apricots, shared constituents include ionone, hexanal, hexenal, hexanol, hexenol, lactones, and terpenic alcohols (Meixia et al., 2004). In Japanese apricot (*Prunus mume* Sieb. et Zucc.), benzaldehyde, isolongifololyl acetate, linalool, butyl acetate, and palmitic acid are the dominant compounds (Miyazawa et al., 2009).

#### Pineapple (*Ananas comosus*)

Pineapple is an important tropical fruit in which the most common VOCs are ethyl 2-methylbutyrate, methyl-2-methylbutyrate, ethyl 2-methylbutanoate, methyl 2-methylbutanoate, methyl hexanoate, ethyl hexanoate, decanal, and 2,5-dimethyl-4-hydroxy-3(2H)-furanone (Montero-Calderón et al., 2010; Wei et al., 2011; Zheng et al., 2012; Žemlička et al., 2013). Other VOCs include furaneol, 3-(methylthio) propanoic acid ethyl ester, 3-(methylthio) propanoic acid methyl ester, δ-octalactone (Zheng et al., 2012), and methyl butanoate (Montero-Calderón et al., 2010). The dominant compounds in pineapple at all growth stages are esters, terpenes, alcohols, 2-ketones, aldehydes, free fatty acids, and γ- and δ-lactones (Steingass et al., 2015).

Pineapple juice comprises mainly esters, decanal, acetic acid, ethyl octanoate, 1-hexanol, γ-octalactone, δ-octalactone, γ-hexalactone, γ-decalactone, and γ-dodecalactone (de Barretto et al., 2013). The major esters are methyl 2-methylbutanoate, methyl 3-(methylthio)-propanoate, methyl butanoate, methyl hexanoate, ethyl hexanoate, ethyl 3-(methylthio)-propanoate, 2,5-dimethyl-4-methoxy-3(2H)-furanone (mesifurane), and 2,5-dimethyl-4-hydroxy-3(2H)-furanone (furaneol) (Elss et al., 2005). In pineapple wine, the main components are ethyl octanoate, ethyl acetate, ethyl decanoate, and 3-methyl-1-butanol (Pino and Queris, 2010).

#### Durian (*Durio zibethinus*)

Durian is a tropical fruit popular in southeast Asia (Chin et al., 2008); it is well-known for its strong aroma and unique taste. Durian produces various VOCs, including esters (ethyl ester and propanoic acid), aldehydes (acetaldehyde), and sulfur compounds (di-ethyl disulfide, di-ethyl trisulfide, and ethyl-propyl disulfide) (Ali et al., 2020).

The VOCs of most major fruits have been identified. The chemical compositions of fruit aromas are listed in Supplementary Table 2.

## Biosynthetic Pathways

### Terpenoid Biosynthetic Pathway

Terpenoids are the largest group of volatile compounds in plants (Muhlemann et al., 2014; Ramya et al., 2018). Based on C_5_ isoprenoid units, terpenoids are classified as C_5_ (hemiterpenes), C_10_ (monoterpenes), C_15_ (sesquiterpenes), C_20_ (diterpenes), C_25_ (sesterpenes), C_30_ (triterpenes), C_40_ (tetraterpenes), and > C_40_ (polyterpenes) (Martin et al., 2003; Ashour et al., 2010). Terpenoids are derived from isopentenyl diphosphate (IPP) or dimethylallyl diphosphate (DMAPP). Terpenoids are classified based on the molar ratio of IPP to DMAPP: monoterpenes (1:1); sesquiterpenes and sterols (2:1); and diterpenes, carotenoids, and polyterpenes (3:1) (Gershenzon and Kreis, 1999). In higher plants, the C_5_ unit is generated by the mevalonic acid (MVA) and 2-c-methylerythritol 4-phosphate (MEP) pathways. In the cytosol, IPP is generated from the mevalonic acid (MVA) pathway, beginning with the condensation of acetyl-CoA (Newman and Chappell, 1999). In plastids, IPP is formed by the MEP pathway, beginning with pyruvate and glyceraldehyde-3-phosphate. In cytosol and plastid, isopentenyl diphosphate isomerase is responsible for the reversible conversion of IPP to DMAPP (Phillips et al., 2008; Jin et al., 2020).

Monoterpenes constitute an important class of aromatic compounds; they are the principal constituents of the scents of flowers and fruits (Croteau et al., 2000). Monoterpenes include limonene, (E)-β-ocimene, myrcene, linalool, and α- and β-pinene (Piechulla and Effmert, 2010; Dudareva et al., 2013; Oyama-Okubo et al., 2013). In plants, monoterpenes are synthesized in plastids (Dudareva and Pichersky, 2000; Chen and Tholl, 2011). IPP is the precursor of geranyl diphosphate (GPP) and geranylgeranyl pyrophosphate (GGPP). Monoterpenes are derived from geranyl diphosphate (GPP, C_10_); GPP synthase catalyzes the conversion of one IPP and one DMAPP molecule to GPP through a head-to-tail condensation reaction (Poulter and Rilling, 1981; Ogura and Koyama, 1998). Sesquiterpenes are synthesized in the cytosol by the MVA pathway. In this pathway, two IPP molecules and one DMAPP molecule are condensed to produce farnesyl diphosphate by farnesyl pyrophosphate synthase. Finally, cytosolic sesquiterpene synthases catalyze the conversion of farnesyl diphosphate to sesquiterpenes (Chen and Tholl, 2011; Monson, 2013). Notably, in snapdragon flowers, sesquiterpenes are synthesized *via* the MEP pathway (Vranová et al., 2013).

**FIGURE 1:**
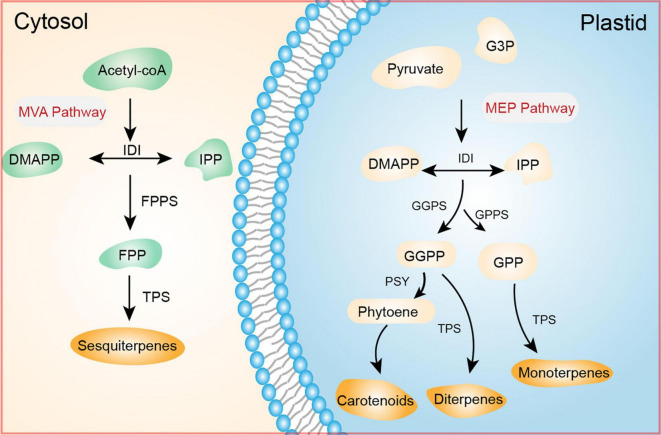
Terpenoid biosynthetic pathway. Terpenoid precursors (acetyl-CoA and pyruvate) enter the MVA pathway in the cytosol to produce sesquiterpenes or the MEP pathway in plastids to generate monoterpenes, diterpenes, and carotenoids. The enzymes and intermediates of both pathways are shown. DMAPP, dimethylallyl diphosphate; FPP, farnesyl diphosphate; FPPS, farnesyl pyrophosphate synthase; GGPP, geranylgeranyl pyrophosphate; GGPS, geranylgeranyl pyrophosphate synthase; GPP, geranyl diphosphate; GPPS, geranyl diphosphate synthase; IDI, isopentenyl diphosphate isomerase; IPP, isopentenyl diphosphate; PSY, phytoene synthase; TPS, terpenoid synthase.

Similar to other isoprenoids, carotenoids share a biosynthetic pathway with mono- and diterpenoids; they are derived from IPP. In plastids (Lichtenthaler et al., 1997), the sequential addition of three IPP molecules to one DMAPP molecule, catalyzed by geranylgeranyl diphosphate synthase, leads to synthesis of the 20-carbon molecule GGPP. The first step in the carotenoid pathway is the condensation of two GGPP molecules to produce phytoene, the primary carotenoid, which is catalyzed by phytoene synthase. Phytoene undergoes several enzymatic reactions to carotenoid compounds (Figure 1; Zhou and Pichersky, 2020).

Terpenoid concentrations in flowers and fruits vary according to developmental stage. For example, in berries, terpenes accumulate during the early stages of development. Terpene compounds synthesized during this stage might be precursors for the final products (Kalua and Boss, 2009). In lavender (*L. angustifolia*), terpenoid quantities are closely related to flower maturity (Li et al., 2019).

Carotenoids are important components of fruit aroma and important precursors of volatile norisoprenoids, which influence the aroma profile of fruits despite their presence at low levels (Watanabe et al., 2009). In some tomato and watermelon varieties, the degradation of carotenoids into lycopene pigment (red) produces geranial, a lemon-scented monoterpene aldehyde (Azulay et al., 2005; Crops et al., 2005). Furthermore, the degradation of β-carotene and lycopene in “Sui hong” papaya fruit results in a pleasant aromatic odor (Jing et al., 2015). In cashew apple (*Anacardium occidentale* L.) juice, the thermal degradation of carotenoids produces an aroma profile of 33 active odor volatiles, such as 1,2,3,5-tetramethylbenzene, naphthalene, and p-xylene (Queiroz et al., 2014). Carotenoids are also involved in floral scent in *Osmanthus fragrans* (Baldermann et al., 2010; Xi et al., 2021). β-ionone, a carotenoid derivative, is produced by the flowering plants *Rosa moschata*, *Thymus vulgaris*, *Viola tricolor*, *Medicago marina*, and *Myrtus communis* (Paparella et al., 2021).

### Phenylpropanoid/Benzenoid Pathway

**FIGURE 2:**
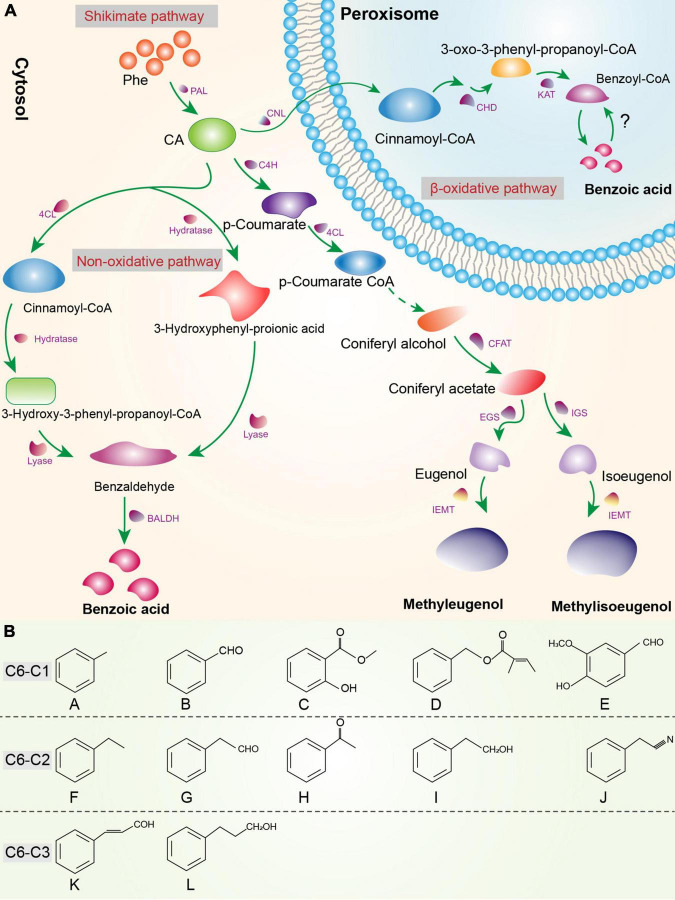
**(A)** Phenylpropanoid and benzenoid biosynthesis. All shikimate pathway enzymes and intermediates in the cytosol and peroxisome are shown. BALDH, benzaldehyde dehydrogenase; CA, *trans-*cinnamate; CHD, cinnamoyl-CoA hydratase/dehydrogenase; CFAT, coniferyl alcohol acyltransferase; C4H, cinnamate 4-hydroxylase; CNL, cinnamate-CoA ligase; 4CL, 4-hydroxycinnamoyl CoA ligase; EGS, eugenol synthase; IEMT, isoeugenol *O*-methyltransferase; IGS, isoeugenol synthase; PAL, phenylalanine ammonia lyase; KAT, 3-ketoacyl thiolase 1.**(B)** Chemical structures of benzenoid (C6–C1) compounds. A, methylbenzene; B, benzaldehyde; C, methyl 2-hydroxybenzoate (methyl salicylate); D, benzyl (£)-2-methyl-2-butenoate (benzyl tiglate); E, 4-hydroxy-3-methoxybenzaldehyde (vanillin). Phenylpropanoid-related (C6–C2) compounds: F, ethylbenzene; G, phenylacetaldehyde; H, acetophenone; I, 2-phenylethanol; J, 2-phenylacetonitrile. Phenylpropanoid (C6–C3) compounds: K (£)-cinnamic aldehyde; L, phenylpropanol.

Phenylpropanoids and benzenoids are the second largest class of plant VOCs (Knudsen and Gershenzon, 2006). Only phenylpropanoids containing a reduced carboxyl group at C_9_ (i.e., aldehydes, alcohols, or alkenes) and/or alkyl addition to hydroxyl groups of the benzyl ring or to the carboxyl group (i.e., ethers and esters) are considered volatiles (Dudareva and Pichersky, 2000). According to the side-chain length, aromatic compounds are classified as phenylpropanoids (C6–C3), phenylpropanoid-related (C6–C2), and benzenoids (C6–C1) (Muhlemann et al., 2014; Lackus et al., 2021; Figure 2B). Phenylpropanoids and benzenoids are produced from the aromatic amino acid phenylalanine (Phe) *via* the shikimate pathway (Maeda and Dudareva, 2012; Yoo et al., 2013). The first step in phenylpropanoid and benzenoid biosynthesis is catalyzed by phenylalanine ammonia-lyase (PAL), the entry-point enzyme of the general phenylpropanoid biosynthetic pathway, which converts Phe to *trans-*cinnamate (CA) (Bate et al., 1994; Boatright et al., 2004; Orlova et al., 2006).

To synthesize phenylpropanoids, CA is hydroxylated by cinnamate 4-hydroxylase (C4H), producing p-coumarate. Consequently, 4-coumaroyl CoA ligase catalyzes the conversion of p-coumarate into p-coumaroyl-CoA (Deng and Lu, 2017). A series of enzymatic reactions is required for the formation of coniferyl alcohol, which is acetylated by coniferyl alcohol acyltransferase and converted to coniferyl acetate. The NADPH-dependent reductases eugenol synthase and isoeugenol synthase (IGS) catalyze the conversion of coniferyl acetate to eugenol and isoeugenol, respectively (Ferrer et al., 2008; Huang et al., 2020). Eugenol and isoeugenol are methylated by isoeugenol *O*-methyltransferases to produce the volatiles methyleugenol and methyl isoeugenol (Gang et al., 2002; Dudareva et al., 2013; Figure 2A).

To synthesize benzenoids, the propyl side chain of CA is shortened by two carbons. Multiple chain-shortening pathways have been proposed: two nonoxidative pathways in the cytosol (CoA-dependent or CoA-independent), and a β-oxidative pathway in peroxisomes (Widhalm and Dudareva, 2015). The CoA-dependent non-oxidative pathway involves the conversion of CA to cinnamoyl-CoA by 4-hydroxycinnamoyl CoA ligase. Thereafter, hydratase catalyzes cinnamoyl-CoA to 3-hydroxy-3-phenyl-propanoyl-CoA, from which benzaldehyde is produced by lyase. In the CoA-independent non-oxidative pathway, CA is converted by hydratase to 3-hydroxyphenyl-propionic acid, which is reduced by lyase to benzaldehyde (Widhalm and Dudareva, 2015). The last step is the production of benzoic acid by benzaldehyde dehydrogenase, as characterized in Snapdragon flowers (*Anthirrhinum majus*) (Long et al., 2009) and proposed in petunia flowers (Kim et al., 2019).

The β-oxidative pathway has been described in petunia (*Petunia hybrida*) flowers and Arabidopsis (*A. thaliana*) (Moerkercke et al., 2009; Qualley et al., 2012). The β-oxidative pathway includes shortening of the propyl side chain in three enzymatic steps. First, CA is converted by cinnamate-CoA ligase to cinnamoyl-CoA. The bifunctional cinnamoyl-CoA hydratase/dehydrogenase, which is responsible for two intermediate steps (hydration and oxidation), converts cinnamoyl-CoA to 3-oxo-3-phenyl-propanoyl-CoA (Qualley et al., 2012). Finally, 3-oxo-3-phenyl-propanoyl-CoA is converted by 3-keto-acyl CoA-thiolase to benzoyl-CoA (Moerkercke et al., 2009). Benzoyl-CoA is a precursor of volatile and nonvolatile benzenoids (Lackus et al., 2021). Unfortunately, the steps and several enzymes of the benzenoid pathway are unknown.

The phenylpropanoid and benzenoid biosynthetic pathways compete for Phe (opposite relationship). For example, in *Phlox subulata* cultivars, the emission of larger quantities of benzenoids will produce little or no phenylpropanoid volatiles and *vice versa* (Majetic and Sinka, 2013). Because phenylpropanoids and their derivatives are widely distributed in flowers (Ramya et al., 2017) and fruits (Neelam et al., 2020), further investigations of their synthesis mechanisms are warranted.

### Fatty Acid Derivatives Pathway

**FIGURE 3:**
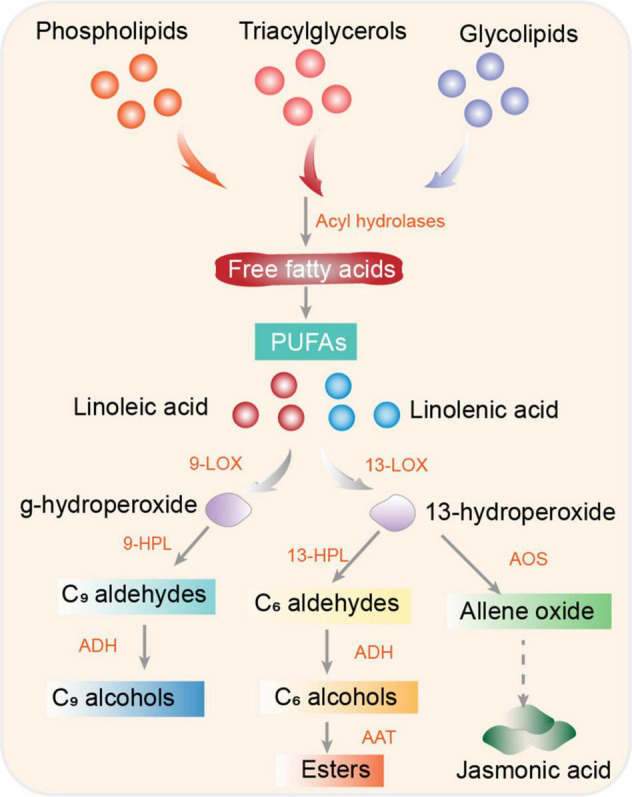
Fatty acid derivative biosynthetic pathway. Fatty acid precursors (linoleic and linolenic acids) enter the LOX pathway and are converted to 9- and 13-hydroperoxide, which are oxidized and converted to volatiles by hydroperoxide lyase (HPL) and alcohol dehydrogenase (ADH). AAT, alcohol acyltransferase; ADH, alcohol dehydrogenase; AOS, allene oxide synthase; HPL, hydroperoxide lyase; LOX, lipoxygenase; PUFAs, polyunsaturated fatty acids.

Fatty acids are the major precursors of volatile compounds in floral scents and fruit aromas; they are catabolized *via* the lipoxygenase (LOX) pathway (Sanz et al., 1997). The LOX pathway is important for the production of aromatic compounds. Polyunsaturated fatty acids—linoleic (C18:2) and linolenic (C18:3) acids—are the main substrates of LOX in plant tissues (Espino-Díaz et al., 2016). Linoleic and linolenic acids are produced from phospholipids, triacylglycerols, and glycolipids by acyl hydrolases. Soluble cytosolic LOXs are divided into two major subfamilies: 9- and 13-LOXs (Baysal and Demirdöven, 2007; Viswanath et al., 2020). LOXs catalyze the addition of oxygen to polyunsaturated fatty acids at the 9 or 12 position, yielding unsaturated 9- or 13-hydroperoxides. Thereafter, 9-hydroperoxide lyase and 13-hydroperoxide lyase convert 9- and 13-hydroperoxides to C9 and C6 aldehydes, respectively. These C9 and C6 aldehydes are reduced by alcohol dehydrogenases (ADH) to C9 and C6 alcohols, whereas C6 alcohols are converted into esters by alcohol acyltransferase (Jerković et al., 2019). The divergence of esters depends on substrate availability, enzyme specificity, and ATT gene variation (Sanz et al., 1997; Espino-Díaz et al., 2016). Jasmonic acid can be generated from 13-hydroperoxy through a separated branch *via* production of an unstable epoxide by allene oxide synthase, followed by a series of cyclization reduction reactions (Muhlemann et al., 2014; Ramya et al., 2018; Figure 3).

### Amino Acid Derivatives

Amino acids are precursors for various plant aromatic secondary metabolites (Vogt, 2010), such as aldehydes, alcohols, acids, and esters. There are three main aromatic amino acids in plants, which are the precursors for various secondary metabolites: Phe, tyrosine (Tyr), and tryptophan (Trp). The amino acid Phe is a precursor for multiple functional secondary metabolites: phenylpropanoids, flavonoids, cell wall lignin, anthocyanins, and various other compounds (Tzin and Galili, 2010b; Qian et al., 2019). Chorismate is converted by chorismate mutase (CM) to prephenate as the first step in Phe biosynthesis (Mobley et al., 1999). The conversion of chorismate to Phe (*via* prephenate and arogenate) is catalyzed by prephenate aminotransferase and arogenate dehydratase, respectively (Cho et al., 2007; Yamada et al., 2008; Maeda et al., 2010). Furthermore, plants can produce Phe through a microbe-like phenylpyruvate pathway involving prephenate dehydratase; flux is increased when the entry point to the arogenate pathway is limiting (Yoo et al., 2013). The volatile Tyr has the same biosynthetic pathway as Phe, involving derivation from chorismate and arogenate. The last step is conversion by arogenate dehydrogenase (TyrA) to Tyr (Schenck et al., 2020; Yokoyama et al., 2021). This pathway has been explained in plants such as tobacco (Gaines et al., 1982), sorghum (Connelly and Conn, 1986), and Arabidopsis (Rippert and Matringe, 2002; Rippert et al., 2009). Alternatively, Tyr biosynthesis can involve prephenate conversion to 4-hydroxyphenylpyruvate by prephenate dehydrogenase and possibly TyrA2 (Rippert and Matringe, 2002). Thereafter, 4-hydroxyphenylpyruvate is converted to Tyr by aminotransferases (e.g., 4-hydroxyphenylpyruvate amino transferase) (Tzin and Galili, 2010a).

**FIGURE 4:**
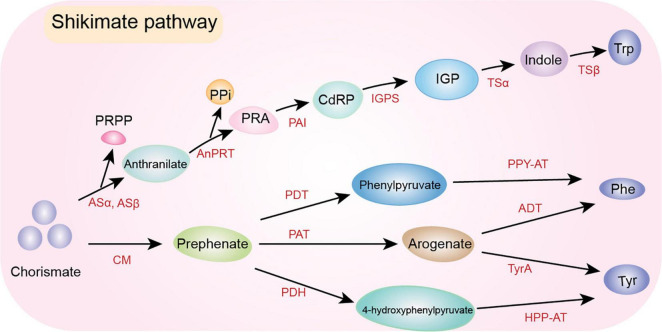
Phe, Tyr, and Trp biosynthetic pathways. Chorismate is the precursor of Phe, Tyr and Trp. Two distinctive pathways starting from the same precursor, Chorismate, where Phe and Tyr share some common intermediates (i.e., prephenate and arogenate) and Trp is produced from a separated branched-chain. ADT, arogenate dehydratase; AnPRT, anthranilate phosphoribosylanthranilate transferase; ASα; ASβ, anthranilate synthase; CdRP, L-(O-carboxyphenylamino)-l-deoxyribulose-5-phosphate; CM, chorismate mutase; HPP-AT, 4-hydroxyphenylpyruvate aminotransferase; IGP, indole-3-glycerol phosphate; IGPS, indole-3-glycerol phosphate synthase; PAI, phosphoribosylanthranilate isomerase; PAT, prephenate aminotransferase; PDH, prephenate dehydrogenase; PDT, prephenate dehydratase; Phe, phenylalanine; PPi, inorganic pyrophosphate; PPY-AT, phenylpyruvate aminotransferase; PRA, phosphoribosylanthranilate; PRPP, phosphoribosyl pyrophosphate; Trp, tryptophan; TSα, tryptophan synthase alpha subunit; TSβ, tryptophan synthase beta subunit; Tyr, tyrosine; TyrA, arogenate dehydrogenase.

The first committed step of volatile Trp biosynthesis is the transfer of an amino group of glutamine to chorismate to produce anthranilate and pyruvate; this is performed by anthranilate synthase (ASα and ASβ) (Tzin and Galili, 2010a). Thereafter, anthranilate phosphoribosylanthranilate transferase (AnPRT) converts anthranilate and phosphoribosyl pyrophosphate into phosphoribosylanthranilate and inorganic pyrophosphate. The next step is conversion of phosphoribosylanthranilate into L-(O-carboxyphenylamino)-L-deoxyribulose-5-phosphate (CdRP); this is performed by phosphoribosylanthranilate isomerase. Furthermore, indole-3-glycerol phosphate synthase catalyzes the conversion of CdRP to indole-3-glycerol phosphate (Li et al., 1995). The last two steps in the Trp biosynthetic pathway are catalyzed by Trp synthase (TS), which has alpha (TSα) and beta (TSβ) subunits. Indole-3-glycerol phosphate is converted by TSα into indole and glyceraldehyde-3-phosphate (α-reaction). Finally, indole is transported to TSβ, which catalyzes its condensation with serine (β-reaction) to generate Trp (Miles, 2001; Weber-Ban et al., 2001; Parthasarathy et al., 2018; Figure 4).

Amino acids undergo deamination or transamination, forming the corresponding α-keto acid, a key intermediate in the conversion of amino acids to volatiles (Gonda et al., 2010; Pott et al., 2019); this reversible step is catalyzed by branched-chain aminotransferases (Klee, 2010). For example, in tomato, six of these enzymes are present in chloroplasts, mitochondria, and the cytoplasm (Kochevenko et al., 2012). Decarboxylation is the most likely route for conversion of α-keto acids to aldehyde volatiles, followed by SDR-mediated synthesis of alcohols (Klee, 2010).

Various synthesis mechanisms of Phe-derived volatiles, such as phenylacetaldehyde and 2-phenylethanol, which contribute to fruit flavors and floral scents have been studied. For instance, in tomato plants, aromatic L-amino acid decarboxylases convert Phe to phenethylamine, the first step in phenylacetaldehyde and 2-phenylethanol synthesis (Tieman et al., 2006). Conversion of phenylacetaldehyde to 2-phenylethanol by phenylacetaldehyde reductases is the last step in the pathway (Tieman et al., 2007). Notably, *P. hybrida* contains a bi-functional decarboxylase/amine oxidase enzyme that directly converts Phe to phenylacetaldehyde (Kaminaga et al., 2006). Additionally, the synthesis of 2-phenylethanol (which has a pleasant fragrance) has been studied in rose (Sakai et al., 2007). Branched-chain alcohols, carbonyls, and esters are produced by metabolism of the amino acids leucine, isoleucine, valine, alanine, and aspartic acid (Heath and Reineccius, 1986; Sanz et al., 1997). Volatile esters are the largest group of volatile compounds produced by fruits. Fruit esters are formed by the esterification of alcohols and acyl CoA, which are derived from fatty acid and amino acid metabolism. This reaction is catalyzed by alcohol acyltransferase (Sanz et al., 1997). In post-climacteric banana slices, amino acids are converted to branched-chain alcohols and esters by aminotransferases, decarboxylases, and ADH (Sanz et al., 1997). The biosynthesis of higher alcohols is linked to amino acid deamination (Herrero et al., 2006).

## Regulation of Floral and Fruit Volatile Compounds

### Factors Affecting Volatile Organic Compound Emission

#### Floral Scent Volatiles

The emission of floral scents is regulated by time of release, environmental variables (light and temperature), tissue type, and plant status (age, physiological state, and sex). These factors stimulate olfactory communication (Raguso, 2008).

The circadian rhythm controls the emission patterns of floral scents in response to the day/night cycle (Picazo-Aragonés et al., 2020). Diurnal and nocturnal emission patterns have been reported in plant species. Some VOCs are emitted independently of environmental conditions; they are influenced by the endogenous biological clock (Zeng et al., 2017). The floral scents emitted by Myrtaceae species at night attract nocturnal bees (Cordeiro et al., 2019). In *Hoya carnosa*, the circadian rhythm is also expressed during exposure to continuous dark (Fenske and Imaizumi, 2016). The impact of the circadian clock on the release patterns of volatile terpenoids and phenylpropanoids/benzenoids has been reported; however, few studies have focused on volatile fatty acid derivatives (Zeng et al., 2017). For example, the circadian clock and light promote the diurnal emission of monoterpenes from orchid (*Phalaenopsis violacea*) flowers (Chuang et al., 2017). The endogenous circadian clock in petunia flower is proposed to regulate the rhythmic emission of volatile phenylpropanoids and benzenoids (Cheng et al., 2016). Moreover, an important fatty acid derivative, jasmonic acid, is controlled by the circadian clock (Zhang Y. et al., 2019).

Volatile compounds are synthesized and emitted from petunia petal limbs during the evening and shortly after anthesis; such emission is drastically decreased when limbs are not supplied with exogenous Phe, and post-pollination-derived ethylene causes the same effect (Dexter et al., 2007). In a single snapdragon flower, the upper and lower petal lobes release high levels of myrcene, ocimene, linalool, and nerolidol (Dudareva et al., 2003; Nagegowda et al., 2008). The cuticle of petunia flowers prevents the release of almost 50% of internal VOCs; a decrease in cuticle thickness influences the synthesis, cellular distribution, and mass transfer resistance of VOCs (Liao et al., 2021).

Floral VOCs respond to changes in environmental conditions, such as temperature, light, water availability, and soil nutrients. As demonstrated by Dötterl et al. (2005), volatile emissions by *Silene latifolia* decrease during hot and dry summer months, whereas low temperatures decrease scent emission in *Pyrola grandiflora* (Knudsen, 1994). Moreover, abiotic stresses influence plant-pollinator and plant-herbivore interactions through different floral VOCs (Campbell et al., 2019).

The developmental stage of flowers affects VOC emission patterns. The changes that occur in petal cells from budding to full blooming, along with the intercellular spaces, modulate VOC evaporation in jasmine (*J. sambac*) flowers (Chen et al., 2020).

### Fruit Aroma Volatiles

Many factors regulate aroma emission by fruits. Fruit genotype influences flavor (Kongor et al., 2016). The final flavor profile is affected by environmental conditions such as climate, sunlight, soil, fruit ripening, harvesting time, and post-harvesting processes (Kongor et al., 2016). Environmental stresses (e.g., temperature and drought) influence fruit metabolism and aromatic compound content (Romero et al., 2021).

The VOC profiles of fruit varieties change according to maturation stage. Terpenoids dominate the aroma profile in some fruits during ripening, such as apple (Liu et al., 2021), apricot (Karabulut et al., 2018), and peach (Wei et al., 2021). In grape, some phenylpropanoids increase with maturation (Kambiranda et al., 2016). Furthermore, fatty acid and amino acid-related compounds increase during the maturation of apple (Liu et al., 2021) and apricot (Karabulut et al., 2018). Therefore, maturation is vital for VOC emission in fruits and affects commercial production.

Pre-harvest factors (water, sunlight, fertilization, and chemical application) influence fruit quality characteristics, including flavor (Ahmed et al., 2013). For example, the use of biofertilizers influences the fruit aroma profile and can improve flavors. Biofertilizer treatment promotes the production of volatile compounds in strawberry (Duan et al., 2021). Treatment of pomegranate and peach with salicylic acid, a natural plant phenolic, improves their quality, including flavor (Erogul and Özsoydan, 2020). Pesticides (e.g., triazole pesticides) can negatively affect fruit flavor compounds (Xiao et al., 2020).

Post-harvest techniques (e.g., chemical applications, radiation, cold, heat, and controlled storage atmospheres) are used to suppress disease and enhance fruit quality during storage (Ahmed et al., 2013). Nitric oxide, salicylic acid, 1-methylcyclopropene, and oxalic acid are used for fruit crops, altering their VOC contents (Bambalele et al., 2021). The application of chemical treatments, individually or in combination, can also alter VOC contents. Controlled atmosphere (CA) treatment of fruit aims to prevent the development of bitter pit disease by altering the aromatic contents (Ahmed et al., 2013). The application of CA treatment in combination with 1-methylcyclopropene to “Fuji” apples stored at room temperature promotes the emission of volatiles (Lu et al., 2018). Ethephon increases and 1-methylcyclopropene decreases the production of volatile compounds in apple (Kondo et al., 2005). Intriguingly, the combination of ethephon with methyl jasmonate decreases or increases VOCs in apple cultivars (Kondo et al., 2005). A combination of methyl jasmonate and ethanol significantly decreases the levels of volatiles in raspberry and enhances the aroma of strawberry (Blanch et al., 2011).

The flavor properties of fruits and their products are affected by ultraviolet radiation in grape (Del-Castillo-Alonso et al., 2021), mango (Wang et al., 2020), strawberry (Warner et al., 2021) and pepper fruit (Ma et al., 2021). Cold storage is used for fruit preservation worldwide. However, chilling injury and aroma loss are problems for commercial fruit production. During cold storage, VOC levels are significantly reduced because of precursor metabolism suppression; this occurs in banana (Zhu et al., 2018b), grape (Matsumoto and Ikoma, 2015), mango (Singh et al., 2004), tomato (Bai et al., 2022), and avocado (García-Rojas et al., 2016). CA storage of fruits is a common preservation technology involving the control of O_2_ and CO_2_ levels. This method increases the levels of some volatile compounds, while decreasing the levels of other volatile compounds. The storage of mango in a high level of CO_2_ reduces the monoterpene and sesquiterpene levels, while increasing the levels of esters and norisoprenoids (Lalel et al., 2003b). High-pressure and decompression technology, ethylene scavengers, ozone (O_3_), active packaging, plasma treatment, high-voltage electrostatic field, and pulse-CA with modified atmosphere preservation have been evaluated for fruit preservation (Fang and Wakisaka, 2021).

Heat treatment is performed after harvesting to control insects, inhibit ripening, and induce resistance to chilling injury (Lu et al., 2007). Heat processing influences fruit aromatic compound contents (e.g., it significant increases off-flavors in watermelon juice) (Aboshi et al., 2020). Drying is used for fruit conservation; it also influences their VOC contents. In banana, ester and aldehyde levels decrease rapidly in response to drying, whereas alcohol levels initially increase then decrease (Saha et al., 2018). The volatile profiles of strawberry subjected to different drying methods have been reported (Abouelenein et al., 2021). Most commercial fruits undergo post-harvest treatments or preservation; however, the effects on fruit aromas are unclear.

### Glycosides, Ethylene, and Enzymes Influencing Volatile Organic Compound Emission

Glycosides control volatile emission by flowers and fruits (Schwab et al., 2015). Glycoside volatiles are odorless and release free aroma volatiles upon hydrolysis by β-glucosidase (Yauk et al., 2014; Schwab et al., 2015). Glycosides enhance the flavor of grape wine and tea; they also modify fruit aroma during maturation, storage, and processing (Birtic et al., 2009; Garcia et al., 2013; Yauk et al., 2014; Ohgami et al., 2015).

Ethylene production accelerates the release of VOCs during fruit ripening. For example, the overexpression of a ripening inhibitor (rin) gene led to decreased ethylene production in tomato fruit, thereby reducing volatile levels. Fruits with antisense aminocyclopropanecarboxylic acid synthase have low levels of several volatiles. Fruits with downregulated phytoene synthase have lower levels of carotenoid-derived volatiles, whereas fruits with antisense pectin methylesterase have a lowered level of methanol (Baldwin et al., 2000).

### Genetic and Epigenetic Regulation of Volatile Organic Compound Biosynthesis

#### Genes

Discovery of important genes in volatile biosynthetic pathways has revealed the molecular mechanisms underlying regulation of floral scent and fruit aroma. Multiple studies have focused on isolating terpenoid-related genes in various plant species. For example, in *Lilium* “Siberia,” three terpenoid-related genes (*LoTPS2*, *LoTPS4* and LoTPS5) have been isolated and characterized. Among them, *LoTPS2* catalyzes (*E, E*)-α-farnesene, *LoTPS4* generates D-limonene and β-myrcene, and *LoTPS5* produces squalene (Abbas et al., 2020a,b). Regarding phenylpropanoids and benzenoids, only the genes responsible for the biosynthesis of phenylacetaldehyde and 2-phenylethanol have been discovered (Picazo-Aragonés et al., 2020) (e.g., *RhPAAS* in rose) (Roccia et al., 2019). Little is known regarding the genes responsible for the synthesis of fatty acid and amino acid derivatives.

Transcriptome analysis is used to identify genes involved in the metabolism of floral scents and fruit aromas. There have been reports of transcriptome and genes underlying the regulation of floral scents and fruit aromas of rose (*R. chinensis* “Old Blush” and *R. odorata* var. *gigantean*) (Guo et al., 2017), orchid (*C. sinense*) (Yang F. X. et al., 2021), orchid (Vanda Mimi Palmer) (Toh et al., 2017), peony (*Paeonia suffruticosa* Andr.) (Zhang et al., 2021), *Lilium* “Siberia” (Hu et al., 2017), water lily (Zhang et al., 2020), carnation (*D. caryophyllus*) (Tanase et al., 2012), lavender (*L. angustifolia*) (Li et al., 2019), jasmine (*J. sambac*) (Chen et al., 2020), *Polianthes tuberosa* (Fan et al., 2018) and the Chinese narcissus (He et al., 2020), banana (Kaur et al., 2021), apple “Ruixue” (Liu et al., 2021), grape (*V. vinifera*) (Sun et al., 2019), mango (Xin et al., 2021), and apricot (Zhang Q. et al., 2019). The identification of sets of genes involved in VOC synthesis will provide insight into the corresponding metabolic pathways.

#### Epigenetics

The formation of VOCs is modulated by DNA methylation, histone protein modifications, and chromatin structure remodeling (Picazo-Aragonés et al., 2020). Cold storage of tomato plant reduces its flavor content; it also causes DNA methylation changes (Zhang et al., 2016). Chromatin-based regulatory mechanisms in petunia flower affect genes involved in phenylpropanoid metabolism, implicating histone acetylation in VOC synthesis (Patrick et al., 2021).

#### Transcription Factors

Numerous transcription factors (TFs) regulate the production of volatile compounds. In petunia (*P. hybrida*) flowers, TFs regulate the phenylpropanoid/benzenoid network. For example, ODORANT1 (ODO1), a member of the R2R3-type MYB family, regulates the synthesis of precursors in the shikimate pathway; it also controls the transcription of entry points to the Phe and phenylpropanoid pathways. Suppression of ODO1 expression reduces the transcript levels of multiple genes in this pathway: DAHPS, EPSPS, PAL, CM, and SAMS (Verdonk et al., 2005). ODO1 activates the ABC transporter promoter (ATP-binding cassette transporter), which is localized to the plasma membrane (Van Moerkercke et al., 2012); it also regulates the emission of volatile compounds (Adebesin et al., 2017). The TF *Lilium hybrid* ODO1 (LhODO1) was isolated from Oriental and Asiatic hybrid lilies; it regulates the production of phenylpropanoids/benzenoids (Yoshida et al., 2018).

EMISSION OF BENZENOIDS II (EOBII), a member of the R2R3-type MYB family, regulates ODO1 and activates the promoter of the biosynthetic gene isoeugenol synthase (Spitzer-Rimon et al., 2010; Colquhoun et al., 2011a; Van Moerkercke et al., 2011). Petunia EOBI (R2R3-MYB–like) interacts with regulatory genes; it acts both downstream of EOBII and upstream of ODO1 (Spitzer-Rimon et al., 2012). Numerous genes in the shikimate and phenylpropanoid pathways [5-enolpyruvylshikimate-3-phosphate synthase (EPSPS), 3-deoxy-D-arabinoheptulosonate 7-phosphate synthase (DAHP synthase), chorismate synthase, CM, arogenate dehydratase, and PAT), and scent-related genes (PAL, isoeugenol synthase, and benzoic acid/salicylic acid carboxyl methyltransferase] are downregulated by the silencing of EOBI expression (Spitzer-Rimon et al., 2012). The interaction between PhERF6 and EOBI negatively regulates benzenoid biosynthesis in petunia (Liu et al., 2017). Moreover, in petunia flower and tobacco, the overexpression of *PAP1* (R2R3 MYB-type TF) increases the levels of anthocyanins and volatile compounds (phenylpropanoids/benzenoids) (Xie et al., 2006; Zvi et al., 2008). In rose, overexpression of *PAP1* increases the levels of volatile phenylpropanoids/benzenoids and volatile terpenoids (Zvi et al., 2012). Furthermore, MYB4TF represses cinnamate-4-hydroxylase in the phenylpropanoid pathway, influencing the production of phenylpropanoid compounds in petunia (Colquhoun et al., 2011b). In the *Cymbidium* cultivar “Sael Bit,” CsMYB1 positively regulates genes responsible for the synthesis of phenylpropanoids/benzenoids and esters. CsMYB1 is highly expressed in petals and columns, particularly at the fully open flower stage, when compared with sepals and labella (Ramya et al., 2019).

Six TF families are implicated in the regulation of terpenoid biosynthesis: AP2/ERF, ARF, bHLH, bZIP, MYB, and WRKY (Picazo-Aragonés et al., 2020). MYC2 (bHLH family) induces the expression of two sesquiterpene synthase genes (TPS11 and TPS21) in Arabidopsis (Hong et al., 2012). In rose flowers, RhMYB1 is involved in floral scent biosynthesis (Yan et al., 2011). In *Syringa oblata* flowers, two R2R3-MYB TFs are upregulated at the bud and flowering developmental stages; they are involved in the biosynthesis of terpenoids and monoterpenoids (Zheng et al., 2015). In *Hedychium coronarium*, HcMYB3-6 (scent related R2R3-MYB TFs) is highly expressed at full flowering stages, but its expression levels are low in other plant organs (Yue et al., 2015). MYBs from *H. coronarium* and *S. oblata* control the biosynthesis of terpenoid volatile compounds (Ramya et al., 2017). In *H. coronarium*, several HcMYB genes regulate terpenoid and benzenoid biosynthesis (Abbas et al., 2021b); HcARF5 regulates β-ocimene production (Abbas et al., 2021a). Some monoterpenes in sweet osmanthus (*O. fragrans*) are regulated by OfWRKY genes (Ding et al., 2020), and OfERF61 regulates β-ionone biosynthesis (Han et al., 2019). In *Phalaenopsis* orchids, multiple TFs (e.g., PbbHLH4, PbbHLH6, PbbZIP4, PbERF1, PbERF9, and PbNAC1) regulate the expression of *PbGDPS* and its downstream putative monoterpene synthases genes, *PbTPS5* and *PbTPS10* (Chuang et al., 2017). CrWRKY1 positively regulates the biosynthesis of terpenoid indole alkaloid in *Catharanthus roseus* (Suttipanta et al., 2011). Artemisinin, a sesquiterpene, is regulated by AabZIP1 in *Artemisia annua* (Zhang et al., 2015). Linalool synthesis in *Cinnamomum osmophloeum* is controlled by CoWRKY (Lin et al., 2014). The overexpression of CpMYC2 increases the linalool level, while overexpression of CpbHLH13 increases the β-caryophyllene level, in wintersweet (*Chimonanthus praecox* L.) flowers (Aslam et al., 2020).

Several TFs linked to fruit VOCs have been characterized. For example, in wine grape, VviWRKY40 regulates monoterpenoid glycosylation (Li et al., 2020). E-geraniol synthesis is positively regulated by CitERF71 in sweet orange (Li et al., 2017). In ripe kiwifruit (*Actinidia arguta*), NAC and ETHYLENE-INSENSITIVE3-like TF binding sites in the terpene synthase-1 protein (*AaTPS1*) promoter activate its expression; the absence of NAC (AaNAC2, AaNAC3 and AaNAC4) reduces the *AcTPS1* transcript and protein levels, as well as the monoterpene volatile content (Nieuwenhuizen et al., 2015). Further studies of TFs will provide insights into the mechanisms underlying regulation of fruit aroma production. The major TFs that regulate VOCs are listed in Table 2.

**TABLE 2:** Major transcription factors that regulate VOC production.

Transcription factor	Compounds	Species	References
ODO1 (MYB)	Benzenoids	Petunia (*P. hybrid)*	Verdonk et al., 2005
EOBI (MYB)	Phenylpropanoids/Benzenoids	Petunia (*P. hybrid)*	Spitzer-Rimon et al., 2012
EOBII (MYB)	Phenylpropanoids/Benzenoids	Petunia (*P. hybrid)*	Spitzer-Rimon et al., 2010; Colquhoun et al., 2011a; Van Moerkercke et al., 2011
PhMYB4	Phenylpropanoids	Petunia (*P. hybrid)*	Colquhoun et al., 2011b
PAP1 (MYB)	Phenylpropanoids/Benzenoids	Petunia (*P. hybrid)*	Xie et al., 2006; Zvi et al., 2008
PhERF6	Benzenoids	Petunia (*P. hybrid)*	Liu et al., 2017
RhMYB1	Involved generally in floral scent biosynthesis	Rose (*R. hybrida)*	Yan et al., 2011
LhODO1 (MYB)	Phenylpropanoids/Benzenoids	Lilies (*Lilium spp.)*	Yoshida et al., 2018
HcMYB3- 6	Involved generally in floral scent production	Butterfly ginger (*H. coronarium*)	Yue et al., 2015
HcMYB	Terpenoids and benzenoids	Butterfly ginger (*H. coronarium)*	Abbas et al., 2021b
HcARF5	β-ocimene	Butterfly ginger (*H. coronarium*)	Abbas et al., 2021a
MYC2	Sesquiterpenes	Arabidopsis (*A. thaliana*)	Hong et al., 2012
CsMYB1	Phenylpropanoids/Benzenoids and esters	*Cymbidium* cultivar “Sael Bit” (*Cymbidium goeringii)*	Ramya et al., 2019
PbbHLH4, PbbHLH6, PbbZIP4, PbERF1, PbERF9, and PbNAC1	Monoterpenes	The Beautiful Phalaenopsis *(Phalaenopsis bellina)*	Chuang et al., 2017
CpMYC2	Monoterpenes (linalool)	Wintersweet (*C. praecox* L.)	Aslam et al., 2020
CpbHLH13	Sesquiterpene (β-caryophyllene)	Wintersweet (*C. praecox* L.)	Aslam et al., 2020
OfWRKY	Monoterpenes	Sweet osmanthus (*O. fragrans*)	Ding et al., 2020
OfERF61	β-ionone	Sweet osmanthus (*O. fragrans*)	Han et al., 2019
CrWRKY1	Terpenoid indole alkaloid	Madagascar periwinkle (*C. roseus*)	Suttipanta et al., 2011
CoWRKY	Linalool	*C. osmophloeum*	Lin et al., 2014
AabZIP1	Sesquiterpene (artimisinin)	Wormwood (*A. annua*)	Zhang et al., 2015
VviWRKY40	Monoterpenoid glycosylation	Wine grapes (*V. vinifera*)	Li et al., 2020
CitERF71	E-geraniol	Sweet orange (*C. sinensis*)	Li et al., 2017
AaNAC2, AaNAC3, AaNAC4	Monoterpene	kiwifruit (*A. arguta*)	Nieuwenhuizen et al., 2015

## Perspectives

Studies of floral and fruit aromas have generated extensive information concerning their volatile compounds, functions, biosynthesis, and regulation. Moreover, new techniques and methods have accelerated the discovery of VOCs and their synthetic enzymes, genes, and TFs, thereby enhancing the broader understanding of VOC biosynthesis in plants. However, several unresolved issues warrant further investigation.

The aromas of flowers and fruits serve as signals to pollinators or fruit eaters; however, most horticultural varieties and cultivars are selected by human preference. The identification of VOCs relevant to human sensory preference is important to ensure that consumer demand is met. Additionally, biotechnological modification of the aromatic characteristics of plants or engineering of synthesis pathways in microbial cell factories could increase aromatic metabolite production for commercial exploitation.

Studies of the composition, synthesis, and regulation of floral scents and fruit aromas have focused on major plants, such as petunia (floral scents), tomato, apple (fruit aromas), and model plants. Little information is available concerning other aromatic plant species, possibly because of scarce genome information and difficulties with genetic transformation. The rapid development of genomic techniques, particularly gene editing, enables research regarding genome functions and regulatory mechanisms in a range of plant species. Flowers differ from edible fruits and may be less restricted by concerns involving public opinion and governmental regulation. Thus, genome editing can be applied to produce flowering plants with novel aromas and phenotypes, as well as improved sensory properties.

Most studies concerning the functions of fruit VOCs have focused on the biological activities of VOCs against microorganisms and insects, or on the attraction of animal dispersers; investigations of flower VOCs have focused on pollinator attraction. However, the effects of flower and fruit VOCs on human health are unknown. While VOCs improve fruit/flower edible/aromatic qualities, they also have potential for improving human physical and mental health. The utility of flower fragrances and fruit aromas as natural adjuvant therapies for chronic diseases or other health problems warrants further research.

## Author Contributions

SM and BJ led the writing of this manuscript, contributed in the writing and reviewing for the manuscript. YW draw the figures. WZ reviewed the manuscript. All authors contributed to the article and approved the submitted version.

## Conflict of Interest

The authors declare that the research was conducted in the absence of any commercial or financial relationships that could be construed as a potential conflict of interest.

## Publisher’s Note

All claims expressed in this article are solely those of the authors and do not necessarily represent those of their affiliated organizations, or those of the publisher, the editors and the reviewers. Any product that may be evaluated in this article, or claim that may be made by its manufacturer, is not guaranteed or endorsed by the publisher.

## Footnotes

1
https://www.statista.com/statistics/264001/worldwide-production-of-fruit-by-variety
