# Supplementary material for: Floral Scents and Fruit Aromas: Functions, Compositions, Biosynthesis, and Regulation
Source: Front Plant Sci. 2022 Mar 10;13:860157. doi: 10.3389/fpls.2022.860157 (PMC8961363; doi:10.3389/fpls.2022.860157)
Supplement: Supplementary file 1 [file Table_1.docx]

**Supplementary Table 1. Chemical compositions of floral scents.**

| **Compound group** | **Compounds** | **Species** | **References** |
| --- | --- | --- | --- |
| Monoterpenoids | Myrcene | Rose (*Rosa damescena*) | (Kiralan, 2015) |
|  |  | Daffodil (*Narcissus pseudonarcissus*) | (Li *et al*., 2018) |
|  |  | Hyacinths (*Hyacinthus* spp.) | (Wang *et al*., 2013) |
|  | Linalool | Peony (*Paeonia* spp*.*) | (Song *et al*., 2018) |
|  |  | Tulip (*Tulipa* L.) | (Oyama-Okubo and Tsuji, 2013) |
|  |  | Lily (*Lilium sp.*) | (Kong *et al*., 2017) |
|  |  | Jasmine (*Jasminum sambac, J. auriculatum, J. grandiflorum and J. multifloru*) | (Ito *et al*., 2002; Lin *et al*., 2013; Bera *et al*., 2015; Chen *et al*., 2017) |
|  |  | Orchid (Vanda Mimi Palmer) | (Mohd-Hairul *et al*., 2010) |
|  |  | Lavender (*Lavandula angustifolia*) | (Venskutonis *et al*., 1997; Won *et al*., 2009; Tschiggerl and Bucar, 2010; Śmigielski *et al*., 2013) |
|  |  | Rose (*R. damascene*) | (Koksal *et al*., 2015) |
|  |  | Daffodil (*N. tazetta*) | (Melliou *et al*., 2007; Song *et al*., 2007; Chen *et al*., 2013) |
|  | Limonene | Tulip (*Tulipa* L.) | (Oyama-Okubo and Tsuji, 2013) |
|  | Eucalyptol | Tulip (*Tulipa* L.) | (Oyama-Okubo and Tsuji, 2013) |
|  |  | Rose (*R. damascene*) | (Koksal *et al*., 2015) |
|  | Ocimene | Lily (*Lilium sp.*) | (Kong *et al*., 2017) |
|  |  | Daffodil (*N. pseudonarcissus*, *N. papyraceus*, *N. tazetta*) | (Wang *et al*., 2013; Ruíz-Ramón *et al*., 2014; Li *et al*., 2018) |
|  | Trans-β-ocimene | *Osmanthus fragrans* | (Zeng *et al*., 2016) |
|  |  | Tulip (*Tulipa* L.) | (Oyama-Okubo and Tsuji, 2013) |
|  | α-Pinene | Tulip (*Tulipa* L.) | (Naomi Oyama-Okubo and Tsuji, 2013) |
|  |  | Rose (*R. damescena*) | (Kiralan, 2015) |
|  | Borneol | Lavender (*L. angustifolia*) | (Śmigielski *et al*., 2013) |
|  | Geraniol | Rose (*R. damascene*) | (Kiralan, 2015; Sparinska and Rostoks, 2015) |
|  | Geranyl acetate | Rose (*R. damascene*) | (Koksal *et al*., 2015) |
| Sesquiterpenes | β-caryophyllene | Peony (*Paeonia sp.*) | (Song *et al*., 2018) |
|  |  | Carnation (*Dianthus elymaiticus*) | (Azadi and Entezari, 2016) |
|  | Caryophyllene | Tulip (*Tulipa* L.) | (Oyama-Okubo and Tsuji, 2013) |
|  | α-farnesene | Tulip (*Tulipa* L.) | (Oyama-Okubo and Tsuji, 2013) |
|  |  | Jasmine (*J. sambac*) | (Lin *et al*., 2013; Chen *et al*., 2017;) |
|  | β-ionone | Tulip (*Tulipa* L.) | (Oyama-Okubo and Tsuji, 2013) |
| Benzenoids | Benzaldehyde | Tulip (*Tulipa* L.) | (Oyama-Okubo and Tsuji, 2013) |
|  | Acetophenone | Tulip (*Tulipa* L.) | (Oyama-Okubo and Tsuji, 2013) |
|  | benzyl alcohol | Tulip (*Tulipa* L.) | (Oyama-Okubo and Tsuji, 2013) |
|  |  | Jasmine (*J. sambac*) | (Lin *et al*., 2013; Chen *et al*., 2017) |
|  | Methyl salicylate | Tulip (*Tulipa* L.) | (Oyama-Okubo and Tsuji, 2013) |
|  |  | Orchid (*Zygopetalum maculatum*) | (Bera *et al*., 2018) |
|  | Methyl benzoate | Lily (*Lilium sp.*) | (Kong *et al*., 2017) |
|  |  | Carnation (*D. elymaiticus*) | (Azadi and Entezari, 2016) |
|  | Benzyl acetate | Jasmine (*J. sambac*) | (Lin *et al*., 2013; Chen *et al*., 2017) |
|  |  | Orchid (*Z. maculatum*) | (Bera *et al*., 2018) |
|  |  | Orchid (Vanda Mimi Palmer) | (Mohd-Hairul *et al*., 2010) |
|  |  | Daffodil (*N. tazetta*) | (Melliou *et al*., 2007; Chen *et al*., 2013; Ruíz-Ramón *et al*., 2014) |
|  | Odiethylbenzene | Orchid (*Z. maculatum*) | (Bera *et al*., 2018) |
|  | p-diethylbenzene | Orchid (*Z. maculatum*) | (Bera *et al*., 2018) |
| Phenylpropanoids | 2-phenylethylacetate | Orchid (*Z. maculatum*) | (Bera *et al*., 2018) |
|  | Phenylethanol | Orchid (Vanda Mimi Palmer) | (Mohd-Hairul *et al*., 2010) |
|  |  | Peony (*Paeonia sp.*) | (Song *et al*., 2018) |
|  |  | Rose (*Hybrid Rugosa*) | (Sparinska and Rostoks, 2015) |
|  | Methlyeugenol | Rose (*R. damescena*) | (Kiralan, 2015; Koksal *et al*., 2015) |
|  | Eugenol | Rose (*R. damescena*) | (Koksal *et al*., 2015) |
|  |  | Jasmine (*J. sambac*) | (Lin *et al*., 2013; Chen *et al*., 2017) |
|  | Nerol | Peony (*Paeonia sp.*) | (Song *et al*., 2018) |
|  |  | Orchid (*R. gigantea, R. gigantea var. harrisonianum Holtt., Vanda coerulea* and *Dendrobium parishii*) | (Julsrigival *et al*., 2013) |
|  |  | Rose (*H. Rugosa*) | (Sparinska and Rostoks, 2015) |
|  | (R)-citronellol | Peony (*Paeonia sp.*) | (Song *et al*., 2018) |
|  | Geranyl acetone | Tulip (*Tulipa* L.) | (Oyama-Okubo and Tsuji, 2013) |
|  | Decanal | Tulip (*Tulipa* L.) | (Oyama-Okubo and Tsuji, 2013) |
|  |  | Carnation (*D. elymaiticus*) | (Azadi and Entezari, 2016) |
|  | Cis-3-hexenol | Tulip (*Tulipa* L.) | (Oyama-Okubo and Tsuji, 2013) |
|  | Cis-3-hexenyl acetate | Tulip (*Tulipa* L.) | (Oyama-Okubo and Tsuji, 2013) |
|  | 2-hexenal | Tulip (*Tulipa* L.) | (Oyama-Okubo and Tsuji, 2013) |
|  | Octanal | Tulip (*Tulipa* L.) | (Oyama-Okubo and Tsuji, 2013) |
|  | Methyl decanoate | Water lily (*Nymphaea colorata*) | (Kong *et al*., 2017) |
|  | (E)-2-hexenyl hexanoate | Jasmine (*J. sambac*) | (Ito *et al*., 2002) |
|  | Methyl anthranilate | Jasmine (*J. sambac*) | (Ito *et al*., 2002; Lin *et al*., 2013; Chen *et al*., 2017;) |
|  | Nonanal | Orchid (*R. gigantea, R. gigantea var. harrisonianum Holtt., Vanda coerulea* and *Dendrobium parishii*) | (Julsrigival *et al*., 2013) |
|  | 2-pentadecanone | Orchid (*R. gigantea, R. gigantea var. harrisonianum Holtt., Vanda coerulea* and *Dendrobium parishii*) | (Julsrigival *et al*., 2013) |
|  | Nerolidol | Orchid (Vanda Mimi Palmer) | (Mohd-Hairul *et al*., 2010) |
|  | Linalyl acetate | Lavender (*L. angustifolia*) | (Venskutonis *et al*., 1997; Won *et al*., 2009; Tschiggerl and Bucar, 2010; Śmigielski *et al*., 2013; Xiao *et al*., 2017) |
|  | Phenethyl ester | Daffodil (*N. tazetta*) | (Song *et al*., 2007) |
|  | 3-phenylpropyl acetate | Daffodil (*N. tazetta*) | (Melliou *et al*., 2007) |
|  | β-phenethyl alcohol | Hyacinths (*Hyacinthus* spp.) | (Wang *et al*., 2013) |
