# Supplementary material for: Floral Scents and Fruit Aromas: Functions, Compositions, Biosynthesis, and Regulation
Source: Front Plant Sci. 2022 Mar 10;13:860157. doi: 10.3389/fpls.2022.860157 (PMC8961363; doi:10.3389/fpls.2022.860157)
Supplement: Supplementary file 2 [file Table_2.docx]

**Supplementary Table 2. Chemical compositions of fruit aromas.**

| **Volatile group** | **Compounds** | **Species** | **References** |
| --- | --- | --- | --- |
| Esters | [Butyl acetate](https://en.wikipedia.org/wiki/Butyl_acetate) | Apple (*Malus floribunda*) | (Dunemann *et al*., 2009) |
|  |  | Apricot (*P. armeniuca*) | (Chen *et al*., 2006) |
|  |  | Apricot (*P. mume*) | (Miyazawa *et al*., 2009) |
|  |  | Muscadine grape (*Vitisrotundifolia*) | (Lee *et al*., 2016) |
|  | Ethyl acetate | Orange (*Citrus* spp*.*) | (Mirhosseini *et al*., 2007) |
|  |  | Apricot (*Prunus* spp.) | (Guillot *et al*., 2006) |
|  |  | Muscadine grape (*V. rotundifolia*) | (Lee *et al*., 2016) |
|  |  | Pineapple (*Ananas* spp.) | (Pino and Queris, 2010) |
|  | [Ethyl butyrate](https://en.wikipedia.org/wiki/Ethyl_butyrate) | Orange (*Citrus* spp*.*) | (Högnadóttir and Rouseff, 2003; Mirhosseini *et al*., 2007) |
|  | Isoamyl acetate | Banana (*Musa spp.*) | (Zhu *et al*., 2018) |
|  | Hexyl acetate | Strawberry (*Fragariachiloensis*) | (Prat *et al*., 2013) |
|  |  | Apple (*M. floribunda*) | (Dunemann *et al*., 2009) |
|  |  | Apricot (*P. armeniuca*) | (Chen *et al*., 2006; Guillot *et al*., 2006; Wang *et al*., 2011; Gokbulut and Karabulut, 2012;) |
|  |  | Grape (*Vitisrotundifolia*) | (Lee *et al*., 2016) |
|  | Propyl acetate | Grape (*V. rotundifolia*) | (Lee *et al*., 2016) |
|  | (E)-2-hexenyl acetate | Apricot (*P. armeniaca*) | (Wang *et al*., 2011; Gokbulut and Karabulut, 2012) |
|  | (Z)-3-hexenyl acetate | Apricot (*P. armeniaca*) | (Wang *et al*., 2011; Gokbulut and Karabulut, 2012) |
|  | Methyl butanoate | Strawberry (*F. ananassa*) | (Yan *et al*., 2018) |
|  |  | Pineapple (*Ananascomosus*) | (Elss *et al*., 2005; Montero-Calderón *et al*., 2010) |
|  | Ethyl butanoate | Mango (*Mangiferaindica*) | (Pino and Mesa, 2006; Pino, 2012) |
|  |  | Strawberry (*F. ananassa*, *F. chiloensis*) | (Prat *et al*., 2013; Yan *et al*., 2018) |
|  |  | Sweet orange (*C. sinensis*) | (Qiao *et al*., 2008; Kelebek and Selli, 2011) |
|  |  | Pineapple (*Ananas* spp.) | (Wei *et al*., 2011) |
|  | Ethyl hexanoate | Strawberry (*F. ananassa*, *F. chiloensis*) | (Prat *et al*., 2013; Yan *et al*., 2018) |
|  |  | Muscadine grape (*Vitisrotundifolia*) | (Lee *et al*., 2016) |
|  |  | Pineapple (*A. comosus*) | (Elss *et al*., 2005; Wei *et al*., 2011; Zheng *et al*., 2012) |
|  | Ethyl octanoate | Pineapple (*A. comosus*) | (Pino and Queris, 2010; Barretto *et al*., 2013) |
|  | Ethyl decanoate | Strawberry (*F. chiloensis*) | (Prat *et al*., 2013) |
|  |  | Pineapple (*Ananas* spp.) | (Pino and Queris, 2010) |
|  | Ethyl‐2‐methylpropanoate | Mango (*M. indica*) | (Pino and Mesa, 2006; Pino, 2012) |
|  | Ethyl 2‐methylbutanoate | Mango (*M. indica*) | (Pino, 2012) |
| Acids | Hexadecanoic acid | Strawberry (*F. chiloensis*) | (Prat *et al*., 2013) |
|  | Butanoic acid | Banana (*Musa* spp.) | (Zhu *et al*., 2018) |
|  | 3-methyl-butanoic acid | Banana (*Musa* spp.) | (Aurore *et al*., 2011) |
|  | Octanoic acid | Banana (*Musa* spp.) | (Zhu *et al*., 2018) |
|  | Palmitic acid | Apricot (*P. mume*) | (Miyazawa *et al*., 2009) |
| Ketones | Acetoin | Banana (*Musa* spp.) | (Aurore *et al*., 2011) |
|  | β-ionone | Apricot (*P. armeniaca*) | (Chen *et al*., 2006; Guillot *et al*., 2006; Solis-Solis *et al*., 2007; Wang *et al*., 2011; Xi *et al*., 2016) |
|  | (E)‐β‐ionone | Mango (*M. indica*) | (Pino and Mesa, 2006) |
|  | Nootkatone | Orange (*Citrus sinensis*) | (Kelebek and Selli, 2011) |
|  | (E)‐β‐damascenone | Mango (*M. indica*) | (Pino, 2012) |
| Aldehydes | (E)-2-hexenal | Banana (*Musa* spp.) | (Aurore *et al*., 2011) |
|  |  | Apricot (*P. armeniaca*) | (Chen *et al*., 2006; Wang *etal*., 2011) |
|  |  | Grape (*V. vinifera*) | (Aubert and Chalot, 2017; Ripoll *et al*., 2017) |
|  | Benzaldehyde | Peach (*Prunuspersica*) | (Aubert and Milhet, 2007) |
|  |  | Apricot (*P. mume*) | (Miyazawa *et al*., 2009) |
|  |  | Grape (*Vitis* spp.) | (Ripoll *et al*., 2017) |
|  | Decanal | Mango (*M. indica*) | (Pino and Mesa, 2006) |
|  |  | Orange (*Citrus* spp.) | (Högnadóttir and Rouseff, 2003; Mirhosseini *et al*., 2007; Qiao *et al*., 2008) |
|  |  | Grape (*V. rotundifolia*) | (Lee *et al*., 2016) |
|  |  | Pineapple (*A. comosus*) | (Wei *et al*., 2011; Zheng *et al*., 2012; Barretto *et al*., 2013) |
|  | Hexanal | Mango (*M. indica*) | (Pino, 2012) |
|  |  | Banana (*Musa* spp.) | (Aurore *et al*., 2011; Zhu *etal*., 2018) |
|  |  | Apricot (*P. armeniaca*) | (Meixia *etal*., 2004; Chen *etal*., 2006; Gokbulut and Karabulut, 2012) |
|  |  | Grape (*V. vinifera*) | (Aubert and Chalot, 2017) |
|  | Nonanal | Mango (*M. indica*) | (Pino, 2012) |
|  |  | Grape (*V. rotundifolia*) | (Lee *etal*., 2016) |
|  | Octanal | Orange (*Citrus* spp*.*) | (Högnadóttir and Rouseff, 2003; Mirhosseini *etal*., 2007; Qiao *etal*., 2008) |
|  | Pentanal | Avocado (*Persea* spp.) | (Obenland *etal*., 2012) |
|  | (E)‐2‐nonenal | Mango (*M. indica*) | (Pino and Mesa, 2006; Pino, 2012) |
|  | (E,Z)‐2,6‐nonadienal | Mango (*M. indica*) | (Pino and Mesa, 2006) |
|  | (E,Z)‐nonadienal | Mango (*M. indica*) | (Pino, 2012) |
|  | (E)‐2‐nonenal | Mango (*M. indica*) | (Pino, 2012) |
|  | Phenylethanal | Grapes (*V. vinifera*) | (Ripoll *etal*., 2017) |
|  | Neral | Orange (*Citrus* spp*.*) | (Mirhosseini *etal*., 2007) |
|  | Geranial | Orange (*Citrus* spp*.*) | (Mirhosseini *etal*., 2007) |
|  |  | Tomato (*Solanum*spp.) | (Azulay *etal*., 2005; Crops *etal*., 2005) |
| Lactones | 2,5‐dimethyl‐4‐methoxy‐3(2H)‐furanone | Mango (*M. indica*) | (Pino and Mesa, 2006; Pino, 2012) |
|  | 4-methoxy-2,5-dimethyl-3(2H)-furanone | Strawberry (*F. ananassa*) | (Yan *etal*., 2018) |
|  | 2,5-dimethyl-4-hydroxy-3(2H)-furanone | Strawberry (*F. ananassa*) | (Yan *et al*., 2018) |
|  |  | Pineapple (*A. comosus*) | (Wei *et al*., 2011) |
|  | 2,5-dimethyl-4-methoxy-3(2H)-furanone (mesifurane) | Pineapple (*A. comosus*) | (Elss *et al*., 2005) |
|  |  | Strawberry (*F. chiloensis*) | (Prat *et al*., 2013) |
|  | 2,5-dimethyl-4-hydroxy-3(2H)- furanone | Pineapple (*A. comosus*) | (Elss *et al*., 2005) |
|  | γ‐octalactone | Mango (*M. indica*) | (Pino, 2012) |
|  |  | Pineapple (*A. comosus*) | (Barretto *et al*., 2013) |
|  | δ-octalactone | Pineapple (*A. comosus*) | (Zheng *et al*., 2012; Barretto *et al*., 2013) |
|  | γ-hexalactone | Pineapple (*A. comosus*) | (Barretto *et al*., 2013) |
|  |  | Apricot (*Prunus* spp.) | (Zhang *et al*., 2008) |
|  | δ-undecalactone | Apricot (*Prunus* spp.) | (Zhang *etal*., 2008) |
|  | γ-decalactone | Pineapple (*A. comosus*) | (Barretto *etal*., 2013) |
|  |  | Apricot (*Prunus* spp.) | (Guillot *etal*., 2006; Solis-Solis *etal*., 2007; Wang *etal*., 2011; Xi *etal*., 2016) |
|  |  | Strawberry (*F. chiloensis*) | (Prat *et al*., 2013) |
|  | γ-dodecalactone | Pineapple (*A. comosus*) | (Barretto *et al*., 2013) |
| Alcohols | 1-Hexanol | Banana (*Musa spp.*) | (Zhu *et al*., 2018) |
|  |  | Apricot (*P. armeniaca*) | (Gokbulut and Karabulut, 2012) |
|  |  | Grape (*V. vinifera*) | (Ripoll *et al*., 2017) |
|  |  | Pineapple (*A. comosus*) | (Barretto *et al*., 2013) |
|  | (E)-2-hexen-1-ol | Apricot (*P. armeniaca*) | (Gokbulut and Karabulut, 2012) |
|  | (Z)-3-hexenol | Apricot (*P. armeniaca*) | (Gokbulut and Karabulut, 2012) |
|  | Benzyl alcohol | Strawberry (*F. chiloensis*) | (Prat *et al*., 2013) |
|  | 2,3-Butanediol | Banana (*Musa* spp.) | (Aurore *et al*., 2011) |
|  | Phenethyl alcohol | Bulgarian rose | (Won *et al*., 2009) |
|  | 3-methyl-1-butanol | Pineapple (*Ananas* spp.) | (Pino and Queris, 2010) |
|  | 1-octanol | Orange (*Citrus* spp*.*) | (Mirhosseini *et al*., 2007) |
|  |  | Grape (*V. rotundifolia*) | (Lee *et al*., 2016) |
|  | 2-phenylethanol | Grape (*V. vinifera*) | (Ripoll *et al*., 2017) |
|  | 2-propyl-1-pentanol | Grape (*Vitis* spp.) | (Palade and Popa, 2016) |
|  | Ethanol | Apricot (*P. armeniaca*) | (Gokbulut and Karabulut, 2012) |
|  | α-terpineol | Lemon (*Citrus limon*) | (Mahalwal and Ali, 2003) |
| Terpenoids | α‐pinene | Mango (*M. indica*) | (Quijano *et al*., 2007) |
|  |  | Orange (*Citrus* spp*.*) | (Qiao *et al*., 2008)(Mirhosseini *et al*., 2007) |
|  | Myrcene | Mango (*M. indica*) | (Pino, 2012) |
|  |  | Orange (*Citrus* spp*.*) | (Högnadóttir and Rouseff, 2003; Mirhosseini *et al*., 2007; Qiao *et al*., 2008) |
|  | Myrcenol | Grape (*V. rotundifolia*) | (Lee *et al*., 2016) |
|  | δ‐3‐carene | Mango (*M. indica*) | (Quijano *et al*., 2007; Pino, 2012) |
|  | Limonene | Mango (*M. indica*) | (Pino, 2012) |
|  |  | Lemon (*Citrus limon*) | (Ayedoun *et al*., 1996; Mahalwal and Ali, 2003; Zhong *et al*., 2014) |
|  |  | Orange (*Citrus* spp*.*) | (Högnadóttir and Rouseff, 2003; Mirhosseini *et al*., 2007; Kelebek and Selli, 2011; Herrera *et al*., 2016) |
|  |  | Apricot (*P. armeniaca*) | (Chen *et al*., 2006; Guillot *et al*., 2006; Solis-Solis *et al*., 2007) |
|  |  | Grape (*V. rotundifolia*) | (Lee *et al*., 2016) |
|  | Linalool | Mango (*M. indica*) | (Pino, 2012) |
|  |  | Strawberry (*F. ananassa*, *F. chiloensis*) | (Prat *et al*., 2013; Yan *et al*., 2018) |
|  |  | Orange (*Citrus* spp*.*) | (Högnadóttir and Rouseff, 2003; Mirhosseini *et al*., 2007; Qiao *et al*., 2008; Kelebek and Selli, 2011) |
|  |  | Apricot (*P. armeniaca*) | (Chen *et al*., 2006; Guillot et al., 2006; Miyazawa*et al*., 2009; Solis-Solis *et al*., 2007) |
|  |  | Peach (*P. persica*) | (Wang *et al*., 2009) |
|  |  | Grape (*V. vinifera*) | (Palade and Popa, 2016; Ripoll *et al*., 2017) |
|  | α‐phellandrene | Mango (*M. indica*) | (Quijano *et al*., 2007) |
|  |  | Lemon (*Citrus limon*) | (Mahalwal and Ali, 2003) |
|  | Terpinolene | Mango (*M. indica*) | (Quijano *et al*., 2007) |
|  | β-ocimene | Mango (*M. indica*) | (Zakaria *et al*., 2018) |
|  |  | Grape (*V. rotundifolia*) | (Lee *et al*., 2016) |
|  | Trans β-ocimene | Mango (*M. indica*) | (Zakaria *et al*., 2018) |
|  | Allo-ocimene | Mango (*M. indica*) | (Zakaria *et al*., 2018) |
|  | β‐caryophyllene | Mango (*M. indica*) | (Pino, 2012) |
|  | Valencene | Lemon (*Citrus limon*) | (Mahalwal and Ali, 2003) |
|  | Nerolidol | Strawberry (*F. ananassa*) | (Yan *et al*., 2018) |
|  | T-nerolidol | Lemon (*Citrus limon*) | (Mahalwal and Ali, 2003) |
|  | α-selinene | Lemon (*Citrus limon*) | (Mahalwal and Ali, 2003) |
|  | β-citral | Grape (*V. rotundifolia*) | (Lee *et al*., 2016) |
|  | β-cyclocitral | Apricot (*Prunus* spp.) | (Guillot *et al*., 2006) |
|  | β-citronellol | Grape (*V. rotundifolia*) | (Lee *et al*., 2016) |
|  | Geranyl acetate | Apricot (*Prunus* spp.) | (Zhang *et al*., 2008) |
|  | Menthone | Apricot (*Prunus* spp.) | (Guillot *et al*., 2006) |
|  | γ-terpinene | Orange (*Citrus* spp*.*) | (Mirhosseini *et al*., 2007) |
|  | 3-carene | Orange (*Citrus* spp*.*) | (Mirhosseini *et al*., 2007) |
|  | Camphene | Lemon (*Citrus limon*) | (Mahalwal and Ali, 2003) |
